# Establishing a Malonyl-CoA Biosensor for the Two Model Cyanobacteria *Synechocystis* sp. PCC 6803 and *Synechococcus elongatus* PCC 7942

**DOI:** 10.1021/acssynbio.5c00320

**Published:** 2025-06-30

**Authors:** Ivana Cengic, Elton P. Hudson

**Affiliations:** School of Engineering Sciences in Chemistry, 124629 Biotechnology and Health, Science for Life Laboratory, KTH Royal Institute of Technology, Stockholm 106 91, Sweden

**Keywords:** biosensor, malonyl-CoA, cyanobacteria, inducible promoter, transcriptional repressor, synthetic biology

## Abstract

Malonyl-CoA, produced by the first committed step of fatty acid biosynthesis, is a precursor for many valuable bioproducts, making it an important metabolic engineering target. Here, we establish a malonyl-CoA biosensor for the model cyanobacteria *Synechocystis* sp. PCC 6803 and *Synechococcus elongatus* PCC 7942. The developed biosensor utilizes FapR, a malonyl-CoA-regulated transcriptional repressor from *Bacillus subtilis*, and novel FapR-regulated and cyanobacteria-compatible hybrid promoters for expressing Yfp, the biosensor output reporter. A l-rhamnose-inducible promoter *P*_*rhaBAD*_, characterized in combination with ribosome binding sites of varied strengths, was evaluated for titratable FapR expression. Additionally, the placement and quantity of the FapR-recognized operator within the hybrid promoter was evaluated for its effect on biosensor performance. The optimal operator placement was found to differ for the biosensor variants that achieved maximum reporter expression in the two considered model cyanobacteria. Overall, this biosensor provides new opportunities for further development of cyanobacterial cell factories.

## Graphical abstract

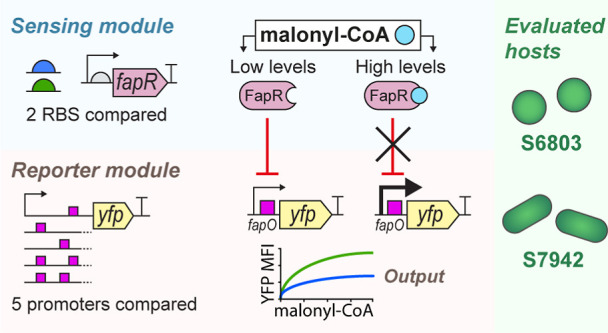

## Introduction

Cyanobacteria are photosynthetic prokaryotes that have gained interest as microbial cell factories for sustainable production of various chemical compounds.^1–3^ Despite a growing number of tools for engineering cyanobacteria,^4–6^ development of metabolite-detecting biosensors has remained relatively unexplored despite their multiapplication potential.^7^

Cell-based biosensors are powerful tools that due to their design flexibility have shown promise in a variety of fields and applications, e.g., in environmental monitoring of pollutants and optimization of chemical bioproduction processes.^8^ Genetically encoded biosensors generally utilize transcription factors, nucleic acids such as riboswitches or aptamers, or protein-based systems such as two-component systems to mediate detection of a molecule of interest.^8–10^ For transcription factor-based biosensors, these are constructed by taking advantage of a metabolite-regulated transcription factor and its recognized *cis*-regulatory operator element.^11–13^ Together they are used to build an interacting sensing and reporter module pair that allows for the detection of the metabolite of interest. The sensing module drives the expression of the transcription factor, whose functional state depends on the intracellular levels of its regulatory metabolite. In the reporter module, the operator is incorporated into a hybrid promoter that drives reporter expression, producing a detectable output when prompted by the action of the transcription factor in the sensing module.

Metabolite biosensors can be used to dynamically regulate biosynthetic pathways, allowing for more efficient use of cellular resources.^11,12,14^ Biosensors coupled to easily screened fluorescent outputs are useful for high-throughput library screening to rapidly identify mutants with improved target metabolite production.^11,15,16^ Similarly, biosensors can facilitate adaptive laboratory evolution experiments by providing a way to screen for and thereby continually enrich outperforming mutants.^11,12,17^ Such tools would significantly speed up cyanobacteria engineering by enabling identification of novel, nonobvious engineering targets.

To date, biosensors in cyanobacteria have mainly been designed to detect environmental signals such as O_2_,^18^ toxins such as heavy metals,^19^ or have relied on assaying cellular health to detect pollutants such as herbicides.^20^ Studies aimed at metabolite detection include development of a FRET-based 2-oxoglutarate biosensor,^21^ a transcriptional repressor-based ammonium biosensor,^22^ and an attempt to establish a transcriptional activator-based H_2_-biosensor.^23^

Malonyl-CoA is an important central metabolite that serves as a precursor for fatty acids, membrane lipids, and other compounds like polyketides and flavonoids.^24,25^ Malonyl-CoA is produced in the first committed step of the fatty acid biosynthesis pathway, wherein carboxylation of acetyl-CoA is catalyzed by acetyl-CoA carboxylase. Intracellular levels of malonyl-CoA are tightly regulated in order for cells to maintain membrane lipid homeostasis.^26^ Consequently, low availability has been implicated as an obstacle to producing malonyl-CoA-derived compounds in cyanobacteria.^27,28^ Improving malonyl-CoA levels is, therefore, an important metabolic engineering goal.

Biosensors for detecting malonyl-CoA have been developed for mammalian cells, *Escherichia coli*, and various yeast strains.^16,29–33^ These biosensors have generally utilized the malonyl-CoA-regulated transcription factor FapR, first characterized in *Bacillus subtilis*.^34,35^ FapR is a transcriptional repressor of genes in fatty acid and phospholipid biosynthesis; it binds to the *fapO*-operator found upstream of regulated genes, thereby controlling flow through these resource-intensive pathways based on malonyl-CoA availability.^26,34,35^ Binding of malonyl-CoA to FapR triggers a conformational change within the repressor, causing it to dissociate from *fapO* and thereby enabling expression of its regulon.^35^ This allows the cell to adjust to and consume detected increases in intracellular malonyl-CoA.

In this study, the FapR-*fapO* system from *B. subtilis* was used to establish a biosensor for detecting the key metabolite malonyl-CoA in the two unicellular, freshwater model cyanobacteria *Synechocystis* sp. PCC 6803 (hereafter S6803) and *Synechococcus elongatus* PCC 7942 (hereafter S7942). This required construction and evaluation of compatible sensing and reporter modules (Figure 1), wherein the former drives expression of the malonyl-CoA-regulated transcriptional repressor FapR and the latter uses a FapR-regulated hybrid promoter to control expression of a Yfp-reporter. The S6803 and S7942 cyanobacteria strains were selected as hosts due to the extensive work that has been done in these model strains to optimize their bioproduction of fatty acid-derived compounds.^2,36–38^ Establishing a malonyl-CoA biosensor could help build upon this already acquired knowledge to further improve yields. Additionally, a working biosensor in the S7942 model strain could be functional in other genetically similar and faster growing *Synechococcus elongatus* variants of interest, specifically *Synechococcus elongatus* UTEX 2973 (hereafter UTEX 2973), *Synechococcus elongatus* PCC 11801 (hereafter S11801), and *Synechococcus elongatus* PCC 11802 (hereafter S11802).^39–41^

**Figure 1:**
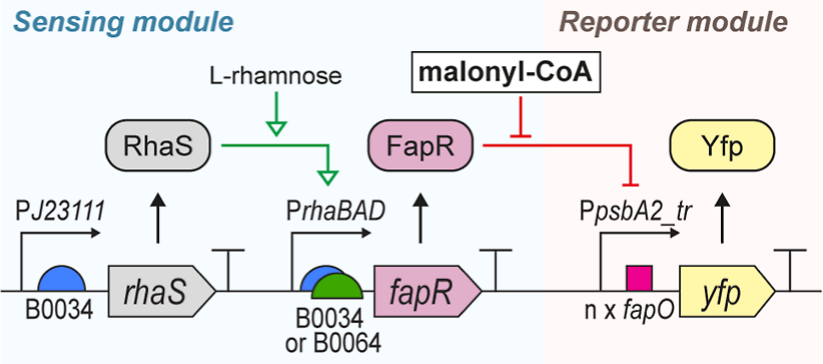
General schematic of the parts and mechanism of the malonyl-CoA biosensor established in this study. The sensing module supports inducible expression of the malonyl-CoA-regulated transcriptional repressor FapR; the inducible expression is driven by *P*_*rhaBAD*_ and its coupled l-rhamnose-regulated activator RhaS. Two different RBS were tested in combination with *P*_*rhaBAD*_, namely, B0034 and B0064. The reporter module drives expression of a Yfp-reporter upon increased intracellular malonyl-CoA levels, since malonyl-CoA blocks the transcriptional repression carried out by FapR onto the Yfp-expressing hybrid promoter (*P*_*psbA2_tr*_ _*-fapO*_). Five different hybrid promoters were tested for driving FapR-controlled Yfp-output.

## Results and Discussion

### *P*_*rhaBAD*_ Enables Inducible and Titratable Expression in S6803 and S7942

Optimizing intracellular FapR levels is important for biosensor performance.^29^ Too much FapR can result in low sensor sensitivity and a high minimum detection limit, while too low levels can cause high basal expression and quick sensor saturation. An inducible promoter would allow for adjustable FapR expression levels (Figure 1).

The l-rhamnose-inducible *rhaBAD* promoter (*P*_*rhaBAD*_) from *E. coli* supports inducible expression in S6803 ^42^ but has not yet been characterized in S7942 or any closely S7942-related cyanobacteria strain. The full-length *P*_*rhaBAD*_ features three different operator sites; these enable the binding of the cAMP receptor protein (CRP), the l-rhamnose controlled RhaS transcriptional activator, and the primary sigma 70 factor in *E. coli* RNA polymerase (RpoD).^42^ While the CRP binding site is necessary for full *P*_*rhaBAD*_ induction in *E. coli*,^43,44^ its omission has been shown to cause no discernible difference to promoter function in S6803.^42^ Additionally, a recent interlaboratory reproducibility study of *P*_*rhaBAD*_ performance in S6803 used a promoter version that lacks the CRP binding site.^45^ Therefore, to minimize the number of nonessential regulatory sequences in the parts evaluated for use in the biosensor constructs in this study, the truncated *P*_*rhaBAD*_ version lacking the CRP binding site was selected for evaluation in both S6803 and S7942. The truncated *P*_*rhaBAD*_ was combined with ribosome binding sites (RBS) of varying expected strengths and evaluated using a Yfp-reporter in both S6803 and S7942 (Figure 2a,d). The selected RBS belong to the BioBrick Registry of Standard Biological Parts and have previously been ranked in terms of strength in S6803: B0034 (100%) > B0064 (∼30%) > B0032 (∼10%).^46^ The transcriptional *P*_*rhaBAD*_-activator RhaS was expressed from the BioBrick BBa_J23111 promoter (*P*_J23111_) combined with RBS-B0034 (Figure 2a,d), a combination shown to support medium strength expression in S6803 and the closely S7942-related UTEX 2973.^39,47^ Note that throughout this study, all S6803 constructs were expressed from the RSF1010-based pPMQAK1-T replicative vector (unless otherwise noted),^47^ while all S7942 constructs were genomically integrated into NS1 (Synpcc7942_2498 locus). Genomic integration was preferred for S7942 due to the previous in-lab experience of genetic instability, via plasmid loss, when using RSF1010-based pPMQAK1-T replicative vectors in this host.

**Figure 2:**
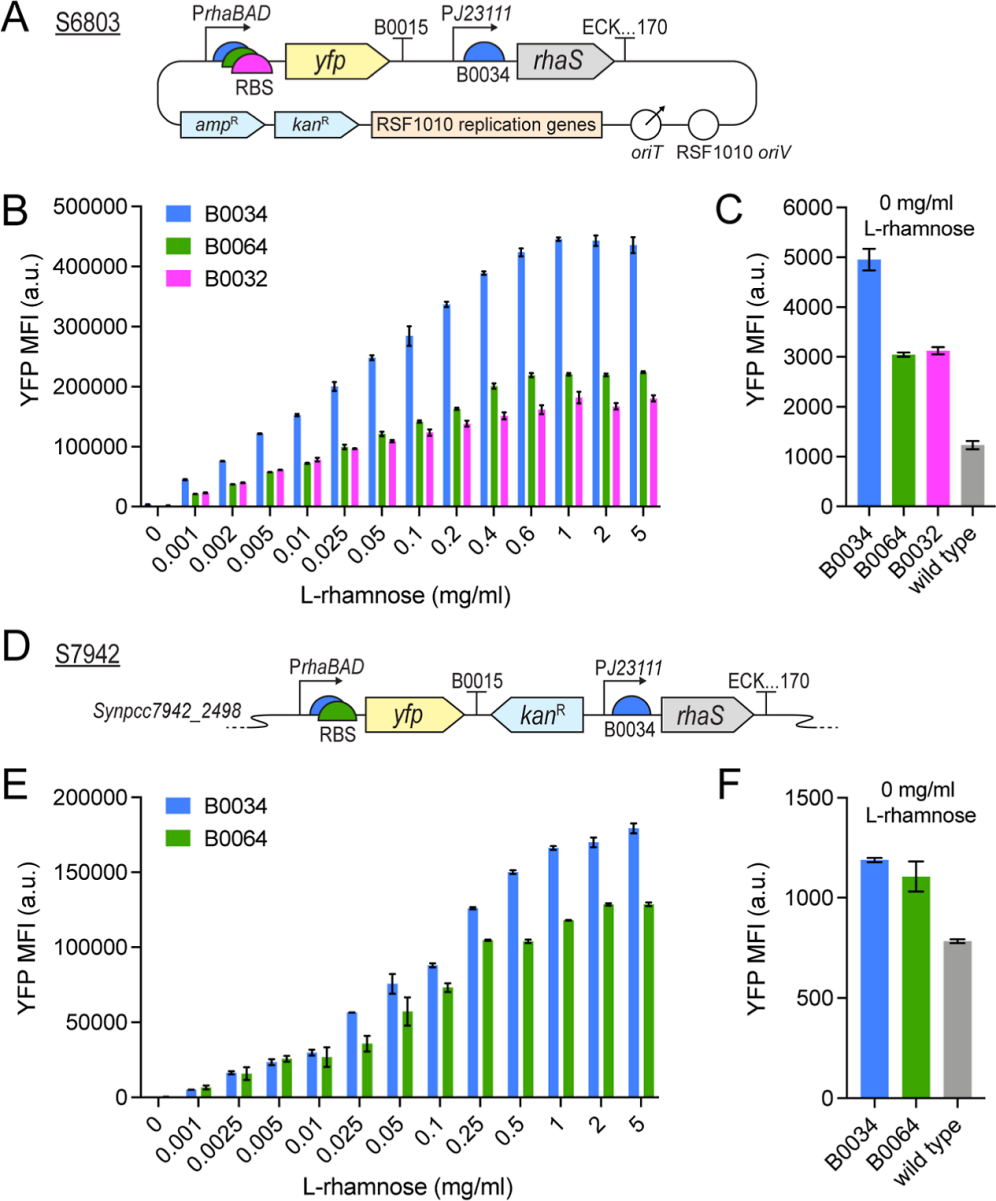
Evaluating the l-rhamnose-inducible *P*_*rhaBAD*_ constructs in (A–C) S6803 and (D–F) S7942 using a Yfp-reporter. (A) Schematic of the replicative pPMQAK1-P_*rhaBAD*_ constructs, with three RBS variants, evaluated in S6803. (D) Schematic of the integrative P_*rhaBAD*_ constructs, with two RBS variants, evaluated in S7942. (A,D) ECK.170 stands for terminator ECK120015170. (B,E) Yfp fluorescence levels measured on day three: (B) 68 h for S6803 and (E) 70.5 h for S7942, after induction with 0–5 mg/mL l-rhamnose. Shown Yfp MFI values have been normalized against FITC MFI values obtained for the corresponding wild-type controls, see the methods section for details. (C,F) Non-normalized Yfp fluorescence levels at uninduced conditions (0 mg/mL l-rhamnose) on day three: (C) 68 h for S6803 and (F) 70.5 h for S7942, compared to the corresponding wild-type controls. All data is presented as averages ±SD for biological triplicates (S6803) or duplicates (S7942).

A three-day time-course experiment was performed to evaluate the P_*rhaBAD*_-RBS-Yfp constructs (hereafter named after their featured RBS), when induced at l-rhamnose concentrations ranging from 0 to 5 mg/mL in both S6803 and S7942. The B0032 construct was not evaluated in S7942 due to three repeated failures to obtain a cyanobacterial transformant. Transformation of S7942 with the B0064 construct also yielded few (<10) colonies compared to transformation with the B0034 construct; the discrepancy for these differing transformation efficiencies is unknown.

Induction of P_*rhaBAD*_ was confirmed for all tested constructs in both S6803 and S7942. Yfp fluorescence levels were overall highest at the end of the three-day time-course (Figure 2b,e); however, similar levels were observed already at day two for S6803 (Figure S1a–c) and were relatively stable all throughout for S7942 (Figure S2a,b).

In S6803, the B0034 construct supported the highest Yfp fluorescence levels, while B0064 and B0032 achieved on average 49% and 45%, respectively, of the B0034-values at 68 h (Figure 2b). While B0064 and B0032 performed similarly at low inducer concentrations, B0064 started outperforming B0032 at 0.05 mg/mL rhamnose (Figure 2b). These results differ slightly from the expected relative RBS-strengths as informed by a previous comparison study in S6803,^46^ wherein B0064 allowed for a roughly 3-fold higher fluorescence level compared to that of B0032. This highlights the need to always characterize new promoter–RBS combinations, as changed sequence contexts can affect expression outcomes of already characterized parts.^48^

The Yfp levels supported by the P_*rhaBAD*_ constructs in S6803 increased linearly with the stepwise increase of l-rhamnose, until a plateau was reached (Figure 2b). Induction-saturation in S6803 was reached for B0034 and B0032 at 1 mg/mL l-rhamnose, while 0.6 mg/mL was enough for B0064 (Figure 2b). These saturating l-rhamnose concentrations are lower than what was observed in a previous study where P_*rhaBAD*_ was combined with RBS*,^49^ a synthetic RBS that generally performs better than B0034 in S6803.^46,50^ The Yfp levels during uninduced conditions (0 mg/mL l-rhamnose) in S6803 were the highest for B0034 (Figure 2c); these measured on average 4.6-fold higher than the wild-type autofluorescence throughout the time-course (Figures 2c and S1d). For the weaker B0064 and B0032 constructs, the uninduced Yfp levels measured similarly at ∼2.5-fold higher than the wild-type autofluorescence at day three (Figure 2c), with B0032 showing slightly higher uninduced Yfp levels than B0064 (+0.5-fold) earlier in the time-course (Figure S1d). The source of Yfp fluorescence at uninduced conditions is from the basal P_*rhaBAD*_-RBS expression in addition to any potential background expression from the replicative pPMQAK1-T vector backbone itself. While this study lacks a control, e.g., a vector lacking a promoter, to quantify the contribution of the latter, previous studies have shown that background expression arising from an empty or promoter-less pPMQAK1-T vector backbone itself is low.^47,51^ Therefore, the majority of the observed Yfp fluorescence at uninduced conditions in S6803 is likely due to the basal expression from P_*rhaBAD*_-RBS.

The P_*rhaBAD*_-RBS constructs were also compared to other commonly used promoters in S6803 (see Table S1 and the Supporting Information for promoter comparison construct details and their promoter–RBS sequences). These comparison constructs were either expressed from a replicative pPMQAK1c shuttle vector^52^ or were genomically integrated into the S6803 *psbA1* (*slr1181*) locus. This pPMQAK1c shuttle vector backbone^52^ is almost identical to the pPMQAK1-T vector backbone^47^ used for the P_*rhaBAD*_ constructs, with the differences being that pPMQAK1c has three retained BpiI-sites and encodes for chloramphenicol resistance instead of kanamycin resistance. When compared to the strong *trc* promoter (P_*trc*_),^47,52^ the induced P_*rhaBAD*_-RBS constructs reached comparable Yfp fluorescence levels to a genomically integrated P_*trc*_-B0034 cassette (Δ*psbA1*::P_*trc*_-B0034-Yfp-B0015) (Figure S1e); however, no P_*rhaBAD*_ construct could match P_*trc*_-B0034 expression from a replicative vector (pPMQAK1c-P_*trc*_-B0034-Yfp-B0015), with P_*rhaBAD*_-B0034 reaching the closest at 78% of the P_*trc*_-B0034 strength when induced at the maximum tested 5 mg/mL l-rhamnose. The above tested P_*trc*_ constructs utilize it as a constitutive promoter by not including coexpression of its *lac* repressor (LacI). However, even when coexpressed with LacI, the basal expression from P_*trc*_ in S6803 has been shown to be very high,^52^ impeding its use as an inducible promoter option in S6803. Therefore, P_*rhaBAD*_ provides a more reliable choice of inducible promoter while also allowing for titratable and high maximum expression levels. When compared to the *psbA2* promoter (P_*psbA2*_),^46,47,53^ the lowest tested l-rhamnose concentration (0.001 mg/mL) was enough for all P_*rhaBAD*_-RBS constructs to exceed P_*psbA2*_ expression from a replicative vector (pPMQAK1c-P_*psbA2*_-Yfp-B0015) (Figure S1f). Genomic expression from P_*psbA2*_ (Δ*psbA1*::P_*psbA2*_-Yfp-B0015) or the anhydrotetracycline-inducible P_L22_-RBS* (Δ*psbA1*::P_L22_-RBS*-Yfp-B0015)^50,54^ was matched by the basal expression from P_*rhaBAD*_-B0034 and P_*rhaBAD*_-B0064/B0032, respectively (Figure S1f). Combining P_*rhaBAD*_ with weaker RBS than the ones tested in this study may allow for construction of inducible promoters that allow for more a stepwise induction to reach the strength of these moderate and weaker promoters. Of note is that P*_rhaBAD_* has an advantage over the aTc-inducible L promoter (P_L_) library since l-rhamnose has been shown to be stable in photoautotrophic growth conditions as opposed to the photolabile aTc.^42,54^ Overall, the strongest Yfp levels in S6803 resulted from P_*trc*_-B0034 when expressed from a replicative vector (pPMQAK1c-P_*trc*_-B0034-Yfp-B0015). This is in line with a previous promoter comparison study in S6803 where P_*trc*_-B0034 ranked as the second strongest promoter.^47^ Additionally, expression from the replicative pPMQAK1c shuttle vector allowed for stronger Yfp fluorescence than compared to genomically integrated cassettes; this is likely due to the higher copy number supported by the replicative RSF1010-based vector compared to the overall genome copy number in S6803.^47,55^

In S7942, the B0034 construct also supported the highest Yfp fluorescence levels, with B0064 performing similarly at low l-rhamnose concentrations (<0.01 mg/mL) but only reaching 72% of the B0034-value at the highest tested 5 mg/mL inducer concentration (Figure 2e). The Yfp levels in S7942 showed a gradual increase within the tested range of l-rhamnose concentrations (Figure 2e); however, the increase was less linear than what was observed in S6803 (Figure 2b). Induction saturation was also less pronounced in S7942 compared to S6803; however, the Yfp levels tapered off at 2–5 mg/mL l-rhamnose for both P_*rhaBAD*_ constructs in S7942. The Yfp levels measured during uninduced conditions (0 mg/mL l-rhamnose) in S7942 were similar for B0034 and B0064 at day three (Figure 2f); these measured on average 1.5-fold and 1.3-fold higher than the wild-type autofluorescence, respectively, throughout the time-course (Figures 2f and S2c). The contribution to these Yfp levels from the genomic read-through at the S7942 NS1 integration site (Synpcc7942_2498 locus) was not quantified in this study; however, the P_*rhaBAD*_ constructs were integrated opposite that of the integration site reading direction, hopefully reducing background levels.

The P_*rhaBAD*_-RBS constructs in S7942 were also compared to a constitutive (no LacI coexpression) P_*trc*_-B0034 construct (see Table S1 and the Supporting Information for promoter comparison construct details and its promoter–RBS sequence). Such a P_*trc*_-B0034 has previously been ranked as the third strongest promoter in a promoter comparison study in the closely S7942-related UTEX 2973.^47^ Comparing P_*rhaBAD*_-RBS to the genomic expression from P_*trc*_-B0034 (ΔNS1::P_*trc*_-B0034-Yfp-B0015) showed that neither of the P_*rhaBAD*_ constructs were able to reach comparable values in S7942, with B0034 and B0064 amounting to only 46% or 33% of the P_*trc*_-B0034 strength when induced at the maximum tested 5 mg/mL l-rhamnose, respectively (Figure S2d). However, similarly to S6803, P_*trc*_ has been shown to maintain high basal expression levels even when coexpressed with LacI in S7942,^56^ reducing its usefulness as an inducible promoter also in S7942. P_*rhaBAD*_ therefore provides a new option for a moderate-strength promoter in S7942 that allows for a more strictly controlled induction.

The above presented results show that a wide range of inducible expression levels are possible for the P_*rhaBAD*_-RBS variants evaluated in S6803 and S7942, and there is therefore the possibility to adjust the inducer concentration based on the desired expression strength and intended application. Overall, for both cyanobacteria, the P_*rhaBAD*_-B0034 construct supported the highest maximum induced Yfp levels, while the P_*rhaBAD*_-B0064 construct had the lowest basal expression at uninduced conditions. Since the FapR levels required for optimal malonyl-CoA biosensor performance were still to be determined, it was decided to evaluate P_*rhaBAD*_ combined with either B0034 or B0064 for driving FapR expression within the malonyl-CoA biosensor sensing module. Both combinations were considered in case the observed variations in induced expression strength and basal expression for the B0034 and B0064 variants would cause noticeable differences in biosensor performance.

### FapR-Regulated Hybrid Promoter Performance Depends on the Placement and Quantity of *fapO* Operator Sites

The FapR-regulated hybrid promoter used within the biosensor must be efficiently repressed by FapR at low malonyl-CoA levels and support detectable reporter expression when FapR dissociates at increased malonyl-CoA levels.^29^ Hybrid promoters are built by combining a host-compatible and well-characterized promoter with the operator recognized by the transcription factor used in the sensing module.^13,29^ The consensus *fapO* operator recognized by FapR from *B. subtilis* consists of a conserved 17 bp inverted repeat;^34^ herein, the *fapO* (TTAGTACCTAGTCTTAA) found specifically in the self-regulated *B. subtilis* *fapR* promoter was used.^34,35^ The *psbA2* promoter (P_*psbA2*_) native to S6803 is well-characterized and also functional in the closely S7942-related UTEX 2973 strain.^47,57,58^ Its native function is to drive expression of the D1 subunit of Photosystem II, and transcription from the full-length P_*psbA2*_ is light-dependent and high-light-stimulated.^53,57–59^ However, truncating P_*psbA2*_ at the 5′ end, upstream of its −35 box, uncouples the light control of its transcriptional activity due to removal of a High Light Regulatory 1 sequence.^60^ Additionally, truncating P_*psbA2*_ at the 3′ end retains expression levels while also removing the transcription start site (TSS) for a stabilizing cis-encoded asRNA (PsbA2R) and a regulatory AU-box.^46,61,62^ Therefore, to avoid including these listed regulatory sites, a minimal P_*psbA2*_ variant truncated at both ends (P_*psbA2_tr*_, −39 to +8 of P_*psbA2*_, see Figure 3a) was combined with the *fapO* operator to construct a set of hybrid promoters to evaluate (Figure 3b). The native P_*psbA2*_ RBS AAGGAA,^53,61^ present between +33 and +43 of P_*psbA2*_, was appended at the 3′ end to drive translation (see Figure 3a).

**Figure 3:**
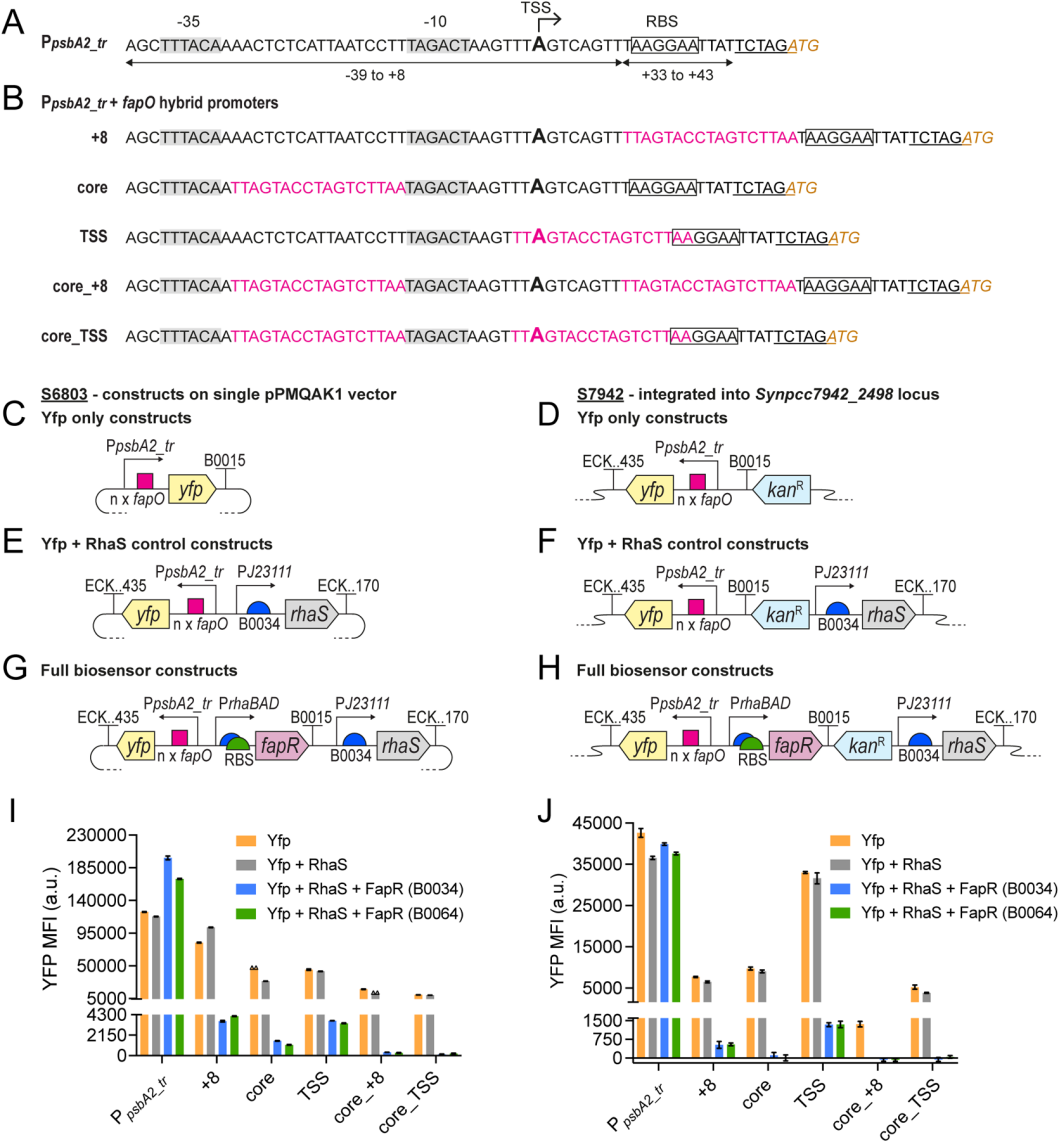
Design and evaluation of FapR-regulated hybrid promoters in S6803 and S7942. (A) Sequence of the truncated P_*psbA2*_ (P_*psbA2_tr*_); bottom arrows and numbers detail the sequences taken from the full-length P_*psbA2*_. The −35 and −10 boxes are shaded, an arrow and larger sized ″A″ marks the mapped TSS, the native P_*psbA2*_ RBS is boxed in, an added XbaI-site used for cloning purposes is underlined, and the start codon is in italic brown text. (B) Sequences of all hybrid promoter variants, combining P_*psbA2_tr*_ and *fapO* (in pink text). The promoter components are detailed as in (A). (C–H) The hybrid promoters were evaluated in several types of constructs; herein, ECK.170 and ECK.435 stand for terminators ECK120015170 or ECK120034435, respectively. Shown schematics include the Yfp only constructs tested in (C) S6803 and (D) S7942, the Yfp and RhaS control constructs tested in (E) S6803 and (F) S7942, and the full biosensor constructs, with sensing modules featuring one of two RBS variants, tested in (G) S6803 and (H) S7942. Note that the S6803 constructs were expressed from the pPMQAK1 replicative vector; for this backbone schematic, see Figure 2a (note that the constructs in (C) have chloramphenicol resistance instead of kanamycin resistance). The S7942 constructs were integrated into the Synpcc7942_2498 locus. (I,J) Yfp fluorescence levels measured after 24 h for (I) S6803 and (J) S7942 strains. Shown Yfp MFI values have been normalized against FITC MFI values obtained for wild-type controls; see the methods section for details. All data is presented as averages ±SD for biological duplicates. Individual duplicate values are shown as open triangles only for the data with nonvisible error bars.

The placement and quantity of operator sites within a hybrid promoter can significantly affect promoter and biosensor performance.^13,29^ Herein, three different *fapO*-positions within the hybrid promoter were assessed (Figure 3b); the first placed *fapO* directly after the 3′ truncation of P_*psbA2_tr*_ at position +8, the second placed *fapO* in the core-region between the −35 and −10 boxes, while the third had *fapO* overlapping with the mapped TSS and RBS of P_*psbA2*_. The third position was guided by the observation that *fapO* and these regions in P_*psbA2*_ shared a sequence similarity. Two additional hybrid promoters were created by combining two of the mentioned *fapO* positions within one promoter, i.e., the core position with either the +8 (core_+8) or TSS-overlap (core_TSS) position. This resulted in five different P_*psbA2_tr‑fapO*_ variants to evaluate (Figure 3b).

The P_*psbA2_tr‑fapO*_ variants were first evaluated with a Yfp-reporter in the absence of FapR expression (Figure 3c,d). In general, the addition of *fapO* to any position in P_*psbA2_tr*_ reduced Yfp expression, and the reduction was most pronounced with two added *fapO* sites (Figure 3i,j). The Yfp fluorescence levels were the highest for the +8-promoter in S6803 (Figure 3i) and the TSS-promoter in S7942 (Figure 3j). These retained 66% and 77% strength, respectively, compared to that of the original P_*psbA2_tr*_. In S6803, the core- and TSS-promoters performed similarly, retaining 38% and 36% strength, respectively (Figure 3i). In S7942, the same applied to the +8- and core-promoters, which retained 18% and 23% strength, respectively (Figure 3j). The double *fapO*-copy promoters (core_+8 and core_TSS) in S6803 and S7942 retained overall less than 14% and 12% strength, respectively (Figure 3i,j). The most diverging result between the two cyanobacteria hosts in this characterization of the P_*psbA2_tr‑fapO*_ variants was for the +8- and TSS-promoters. In previous studies where promoter–RBS combinations have been evaluated in parallel in both S6803 and S7942, or the closely S7942-related UTEX 2973, promoter performance has been shown to overall correlate or differ only slightly between the two hosts,^47^ but examples of more drastically diverging performance have also been described.^63^ One study showed that addition of a ribozyme-based insulator sequence (RiboJ) upstream of an optimized RBS combined with the *conII* promoter (P_*conII*_) resulted in a positive effect on expression levels for two different fluorescent reporters in S6803 but a negative effect when tested in S7942.^63,64^ It is possible that the difference in sequence context upstream of the RBS in the +8- and TSS-variants of the P_*psbA2_tr‑fapO*_ hybrid promoter (Figure 3b) is a reason for the observed difference in promoter function in S6803 and S7942. Additionally, the different 5′ UTR lengths for the +8 (41 nt) and TSS (28 nt) hybrid promoters align better with the median 5′ UTR lengths that have been described for the transcriptomes in S6803 (42 nt or 52 nt)^65,66^ and S7942 (30 nt),^67^ respectively.

Next, it was necessary to assess the potential cross-reactivity between the P_*psbA2_tr‑fapO*_ promoter variants and the P_*rhaBAD*_-activator RhaS from the sensing module. For this, the Yfp constructs were combined with the established RhaS expression cassette (Figure 3e,f). Note that the core_+8-promoter in combination with RhaS was not tested in S7942 due to failure to obtain a S7942 transformant despite three transformation attempts; the reason for this is unknown. In both S6803 and S7942, the addition of RhaS marginally reduced the expression strength of most P_*psbA2_tr‑fapO*_ variants (Figure 3i,j). The biggest reductions in S6803 and S7942 were seen for the core- (−38%) and core_TSS-promoters (−26%), respectively. One exception was the 26% increased expression observed for the +8-promoter in S6803 (Figure 3i). Despite these inconsistent effects, no changes were made to the biosensor designs.

Finally, the full biosensor constructs were completed by combining the different P_*psbA2_tr‑fapO*_-Yfp reporter modules with the complete sensing modules, i.e., FapR expressed by P_*rhaBAD*_-B0034 or P_*rhaBAD*_-B0064 (Figure 3g,h). Since the FapR levels required for optimal malonyl-CoA biosensor performance were still unknown, a first experiment was performed where no l-rhamnose was added to induce FapR expression from P_*rhaBAD*_, thereby assessing the effect from the basal FapR expression levels. It was found that this basal, uninduced FapR expression from the P_*rhaBAD*_-B0034/B0064 sensing modules was enough to promote efficient repression of *yfp* expression from all P_*psbA2_tr‑fapO*_ variants in both S6803 and S7942 (Figure 3i,j). No clear differences were found between the B0034 and B0064 biosensor constructs in either host, indicating that repression of the P_*psbA2_tr‑fapO*_ variants was saturated at the lower basal expression levels from the weaker P_*rhaBAD*_-B0064 sensing module. Hereafter, basal FapR expression from the sensing module was the default, unless otherwise noted. The double *fapO*-copy promoters (core_+8 and core_TSS) were repressed at a minimum 97% and 99% in S6803 and S7942, respectively (Figure 3i,j). Thereafter, the averaged repression for both the B0034 and B0064 biosensor variants in S6803 was 96% for +8, 95% for core, and 92% for TSS (Figure 3i). For S7942, the same averaged repression was 99% for the core, 96% for TSS, and 92% for +8 (Figure 3j). In S6803, the addition of FapR caused increased expression from the control construct P_*psbA2_tr*_ (no *fapO*), as its expression-levels rose by 68% or 44% when combined with the B0034 or B0064 sensing module, respectively (Figure 3i). However, as no such stimulation was seen in S7942 (Figure 3j); this could be due to FapR disrupting regulation of P_*psbA2_tr*_ in its native S6803 host, possibly through interaction with the sequence that is found in both *fapO* and by the TTS in P_*psbA2*_ (i.e., the sequence TTAGT). These results therefore showcase a potential lack of orthogonality between FapR and S6803.

### Biosensor Performance Depends Mainly on the FapR-Regulated Hybrid Promoter in the Reporter Module

The biosensors were next tested for their ability to detect increased malonyl-CoA levels. A common method for forcing intracellular malonyl-CoA accumulation is to subject cells to cerulenin, a β-ketoacyl-ACP synthetase inhibitor.^68,69^ Cerulenin covalently binds to and inhibits FabF and FabH, thereby blocking the malonyl-CoA-consuming condensation steps in fatty acid biosynthesis.^68,69^ Cerulenin has been widely used in proof-of-concept studies for malonyl-CoA biosensors.^29^ Wild-type S6803 and S7942 were subjected to 0–2 μg/mL cerulenin to judge its effect on growth. Clear growth inhibition was found for both strains at 0.5–2 μg/mL cerulenin (Figures 4a and 5a). Based on this, a 0–0.75 μg/mL cerulenin range was selected for evaluating the biosensors, and the resulting low to moderate growth inhibition was expected to translate into a well-distributed malonyl-CoA accumulation range. A two-day time-course experiment was performed to evaluate the biosensor response to cerulenin treatment.

**Figure 4:**
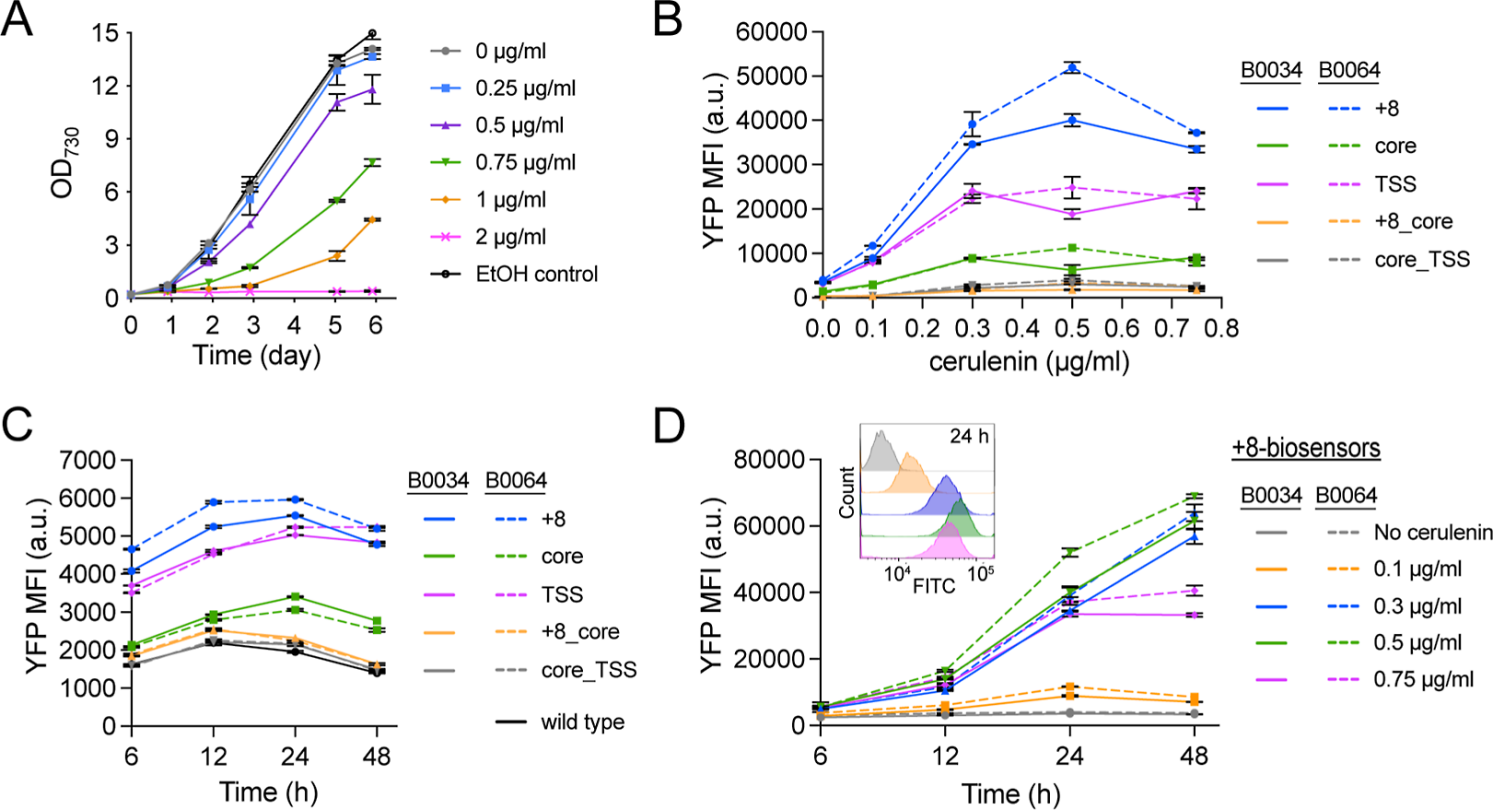
Evaluating the malonyl-CoA biosensor constructs in S6803. (A) Growth curves for S6803 wild-type-grown with 0–2 μg/mL cerulenin. The EtOH control tested the growth effect of the maximum amount of added ethanol (as in 2 μg/mL cerulenin). (B–D) P_*rhaBAD*_-B0034 and P_*rhaBAD*_-B0064 biosensor constructs are represented by solid or dashed lines, respectively. The biosensor variants are further denoted by the name of the hybrid promoter variant used in the reporter module, see Figure 3b for details. (B) Yfp fluorescence levels measured for the full set of malonyl-CoA biosensor variants after 24 h of treatment with 0–0.75 μg/mL cerulenin. Shown Yfp MFI values have been normalized against FITC MFI values obtained for wild-type controls treated with the corresponding cerulenin concentrations, see the methods section for details. (C) Non-normalized basal (no cerulenin added) Yfp MFI levels for the full set of malonyl-CoA biosensor variants throughout a two-day time-course. Values for a wild-type control are also shown. (D) Yfp fluorescence levels measured for the +8-biosensor variants throughout a two-day time-course of treatment with 0–0.75 μg/mL cerulenin; basal (no cerulenin added) values are also shown. Shown Yfp MFI values have been normalized against FITC MFI values obtained for wild-type controls treated with the corresponding cerulenin concentrations, see the methods section for details. The inset figure shows overlaid histograms of the FITC-channel data (excitation 488 nm, emission 525/40 nm) for the P_*rhaBAD*_-B0064 biosensor variant at 24 h (the colors are consistent with the legend in the main figure). All data is presented as averages ±SD for biological duplicates. Nonvisible error bars are smaller than the data symbol.

**Figure 5:**
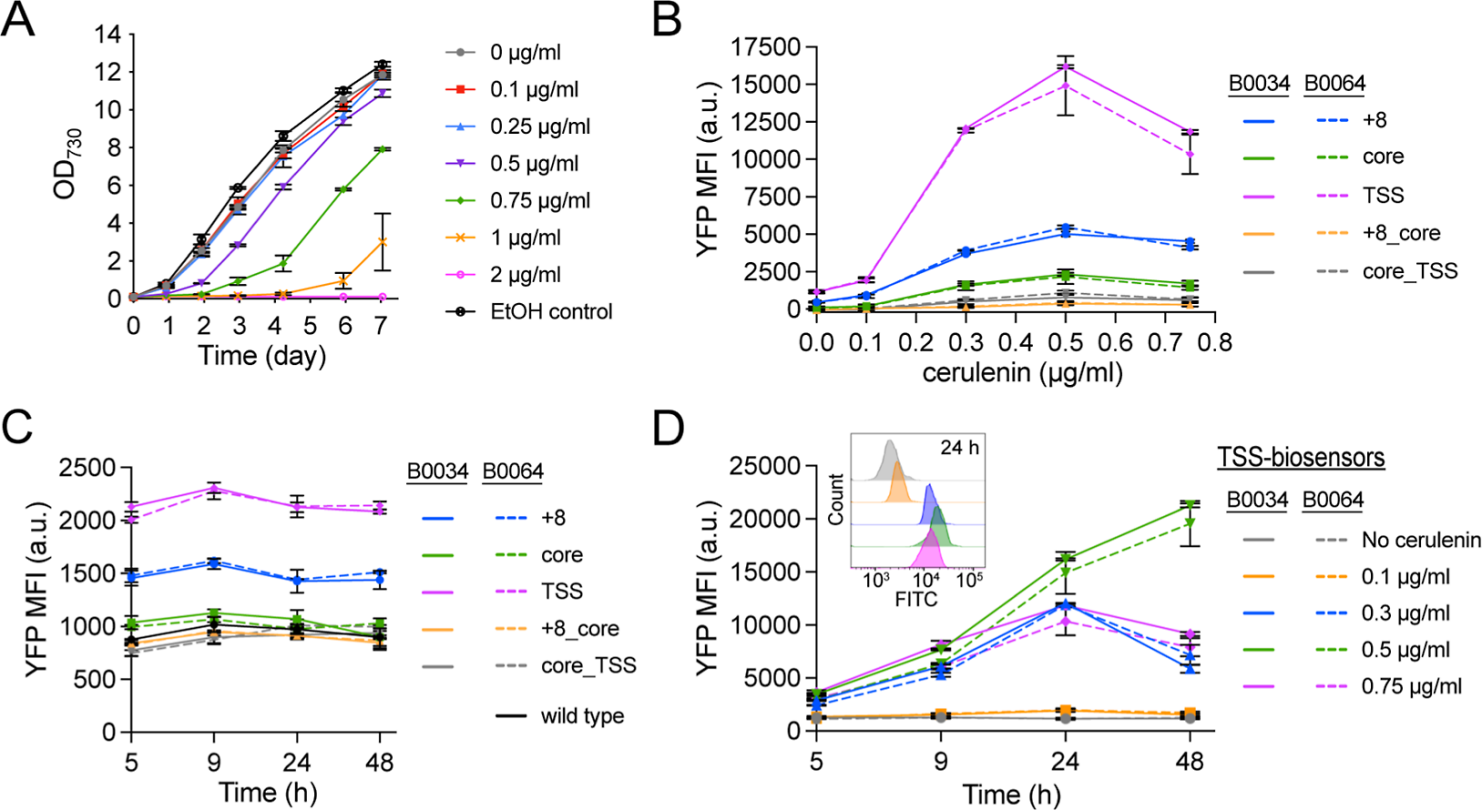
Evaluating the malonyl-CoA biosensor constructs in S7942. (A) Growth curves for S7942 wild-type-grown with 0–2 μg/mL cerulenin. The EtOH control tested the growth effect of the maximum amount of added ethanol (as in 2 μg/mL cerulenin). (B–D) P_*rhaBAD*_-B0034 and P_*rhaBAD*_-B0064 biosensor constructs are represented by solid or dashed lines, respectively. The biosensor variants are further denoted by the name of the hybrid promoter variant used in the reporter module, see Figure 3b for details. (B) Yfp fluorescence levels measured for the full set of malonyl-CoA biosensor variants after 24 h of treatment with 0–0.75 μg/mL cerulenin. Shown Yfp MFI values have been normalized against FITC MFI values obtained for wild-type controls treated with the corresponding cerulenin concentrations, see the methods section for details. (C) Non-normalized basal (no cerulenin added) Yfp MFI levels for the full set of malonyl-CoA biosensor variants throughout a two-day time-course. Values for a wild-type control are also shown. (D) Yfp fluorescence levels measured for the TSS-biosensor variants throughout a two-day time-course of treatment with 0–0.75 μg/mL cerulenin; basal (no cerulenin added) values are also shown. Shown Yfp MFI values have been normalized against FITC MFI values obtained for wild-type controls treated with the corresponding cerulenin concentrations, see the methods section for details. The inset figure shows overlaid histograms of the FITC-channel data (excitation 488 nm, emission 525/40 nm) for the P_*rhaBAD*_-B0064 biosensor variant at 24 h (the colors are consistent with the legend in the main figure). All data is presented as averages ±SD from biological duplicates. Nonvisible error bars are smaller than the data symbol.

For the S6803 biosensor strains, at 24 h of cerulenin treatment, the highest reporter output was observed for the +8-biosensor variants, followed by the TSS- and core-variants (Figure 4b). This pattern matched the strength-rankings of the previously evaluated hybrid promoter and RhaS control constructs (Figure 3i). Reporter outputs for the double *fapO* biosensor variants (core_+8 and core_TSS) were very low (Figure 4b), due to the overall weakness of these hybrid promoters (Figure 3i) and likely stronger repression resulting from double *fapO* operators. The +8-biosensor with the P_*rhaBAD*_-B0064 sensing module performed better than the corresponding P_*rhaBAD*_-B0034 variant; these supported 13- and 11.2-fold induction at 0.5 μg/mL cerulenin, respectively (Figure 4b). The lower basal expression from the P_*rhaBAD*_-B0064 combination compared to P_*rhaBAD*_-B0034 (Figures 2c and S1d) therefore appears to have positively affected the dynamic range of the +8-biosensor; however, such an RBS-dependent effect was not consistently seen for the TSS- or core-biosensor variants in S6803. Induction folds for the TSS- and core-biosensors were similar; they reached an average 7.2- and 7-fold induction across 0.3–0.75 μg/mL cerulenin, respectively. This is despite the TSS-biosensors having an average 2.7-fold higher Yfp-output than the core-biosensors (Figure 4b). Instead, the core-biosensor variants reached a similar dynamic range by benefiting from the lowest basal Yfp fluorescence of all single *fapO*-copy biosensors (Figure 4c). Basal Yfp fluorescence from the +8- and TSS-biosensor variants was similar throughout the experiment, with the +8-biosensor with the P_*rhaBAD*_-B0064 sensing module having the highest overall (Figure 4c).

Time-course data for the +8-biosensor variants in S6803 showed continuously increasing Yfp-output when exposed to 0.3 and 0.5 μg/mL cerulenin (Figure 4d). At 0.75 μg/mL cerulenin, the Yfp-output plateaued after 24 h; it is likely that the more severe effect on cell growth caused by this higher cerulenin concentration also negatively affected biosensor performance. The same described time-course Yfp-output patterns were observed also for the TSS- and core-biosensor variants (Figure S3a,b). The S6803 control strains, containing a reporter module where P_*psbA2_tr*_ lacks *fapO*, also showed increased fluorescence upon treatment with cerulenin (Figure S3c). However, these induction folds were lower than what was observed for the complete *fapO*-featuring biosensors constructs, e.g., at 24 h, the P_*rhaBAD*_-B0064 control strain only had a 1.7-fold increase at 0.5 μg/mL cerulenin. The increased fluorescence for the control strains is likely in part due to higher autofluorescence, a phenotype that can signal cellular health deterioration,^70,71^ and here caused by increasingly toxic cerulenin levels. Similarly, wild-type S6803 also exhibited increased autofluorescence in response to cerulenin (Figure S3d).

For the S7942 biosensor strains, the dose–response curves after 24 h of cerulenin treatment showed the highest Yfp-output for the TSS-biosensor variants, followed by the +8- and core-variants (Figure 5b). This coincided with the TSS hybrid promoter being the strongest in S7942 (Figure 3j), however, with the strength ranking for the core- and +8-promoters being opposite that of the results when evaluated in the Yfp-only constructs (Figure 3j). The weaker performance of the core-promoter in the biosensor context in S7942 could be explained by the core position of its *fapO* operator, as this is the position known to invoke the strongest repression within transcription factor-regulated promoters.^72^ The double *fapO*-copy biosensor variants (core_+8, core_TSS) expectedly supported only weak reporter outputs in S7942 (Figure 5b). No big differences were observed in biosensor performance in S7942 when the P_*rhaBAD*_-B0034 or P_*rhaBAD*_-B0064 sensing modules were used (Figure 5b). The TSS-biosensor variants supported an average 13.4-fold induction at 0.5 μg/mL cerulenin. In contrast, the P_*rhaBAD*_-B0034-variants of the +8- and core-biosensors were 11- and 23.7-fold-induced at 0.5 μg/mL cerulenin, respectively. The high dynamic range for the core-biosensors was again due to having the lowest basal Yfp fluorescence of all single *fapO*-copy biosensors (Figure 5c). Basal Yfp fluorescence was the highest for the top reporter-expressing TSS-biosensors (Figure 5c), thereby decreasing their dynamic range.

Time-course data for the TSS-biosensor variants in S7942 showed continually increasing Yfp-output when treated with 0.5 μg/mL cerulenin (Figure 5d). The output at 0.75 μg/mL of cerulenin decreased after 24 h, likely due to its earlier noted negative effect on cell health and therefore likely biosensor performance. However, reduced biosensor output in S7942 was also found for the lower 0.3 μg/mL of cerulenin after 24 h. It is possible that in contrast to S6803, S7942 was able to adjust to and consume the malonyl-CoA accumulation resulting from 0.3 μg/mL cerulenin. The same described time-course Yfp-output patterns were observed also for the +8- and core-biosensor variants (Figure S4a,b). As in S6803, the P_*psbA2_tr*_ control (no *fapO*) in S7942 showed increased fluorescence when subjected to higher concentrations of cerulenin (Figure S4c), e.g., at 24 h, the P_*rhaBAD*_-B0034 control strain had a 1.6-fold increase at 0.5 μg/mL cerulenin. This is likely partly due to autofluorescence caused by cerulenin-induced cellular stress; however, the autofluorescence phenotype was less pronounced for cerulenin-treated wild-type S7942 (Figure S4d). Therefore, the intracellular effects from cerulenin also appear to have some indirect ability to induce expression from the control non-*fapO* construct in S7942.

### Higher FapR Levels Reduce the Biosensor Yfp-Output at Both Basal and Malonyl-CoA-Induced Conditions

Finally, the top-performing biosensor variants were evaluated at higher FapR levels, achieved by inducing the P_*rhaBAD*_ sensing modules. Based on the earlier characterization of P_*rhaBAD*_ in S6803 and S7942 (Figure 2b,e), inducer concentrations that would give a roughly 5- or 20-fold increase in FapR expression, compared to the basal level, were selected. In both S6803 and S7942, this was found to be 0.001 and 0.005 mg/mL l-rhamnose. The biosensor variants tested under these conditions were the +8-biosensor (P_*rhaBAD*_-B0064-FapR) for S6803 and the TSS-biosensor (P_*rhaBAD*_-B0064-FapR) for S7942. To promote intracellular malonyl-CoA accumulation in these experiments, 0.5 μg/mL cerulenin was used.

In both S6803 and S7942, the Yfp output of the evaluated biosensor variants in response to intracellular malonyl-CoA accumulation decreased with increased FapR levels (Figure 6a,c). In S6803, the averaged Yfp-output for the last two time points (28.5 and 48 h) was 52% and 65% lower when P_*rhaBAD*_-B0064-FapR was induced with 0.001 and 0.005 mg/mL l-rhamnose, respectively. In S7942, the averaged Yfp-output for the last two time points (23 and 46 h) instead showed a 6% and 19% reduction when inducing P_*rhaBAD*_-B0064-FapR at the two previously noted concentrations, respectively. The basal biosensor Yfp fluorescence at no-cerulenin conditions was likewise reduced in both hosts at increased FapR levels (Figure 6a,c). In S6803, the averaged basal Yfp fluorescence for the last two time points (28.5 and 48 h) was 44% and 55% lower during the two listed P_*rhaBAD*_-induction conditions, respectively. In S7942, the averaged basal Yfp fluorescence for the last two time points (23 and 46 h) was reduced by 37% and 60% when P_*rhaBAD*_ was induced with 0.001 and 0.005 mg/mL l-rhamnose, respectively. The effect on growth from induced, higher FapR expression was also evaluated by growing selected strains with the higher tested l-rhamnose concentration (0.005 mg/mL). The induced S6803 and S7942 biosensor cultures reached a 28% and 10% lower final cell density than the uninduced cultures, respectively (Figure 6b,d). Additionally, the induced S6803 biosensor culture appeared more yellow throughout the experiment (not shown). Affected growth was observed only for the l-rhamnose-induced biosensor strains and not for the Yfp + RhaS or wild-type controls, indicating that it was caused by the higher FapR levels. This suggests that FapR likely exhibits unwanted cross-reactivity by potentially disrupting native regulation or exhibiting toxicity within both hosts. Although as the negative growth effect was lower in S7942 than in S6803 (Figure 6b,d), this could be an indication that S7942 is less cross-compatible with FapR than S6803. However, the difference could also be because induction of P_*rhaBAD*_-B0064-FapR with 0.005 mg/mL l-rhamnose in S7942 leads to a lower intracellular concentration of FapR compared to the same P_*rhaBAD*_-induction conditions in S6803, since expression in S7942 is driven from a genomic integration site rather than a higher copy replicative plasmid as in S6803, causing a lower overall negative effect on growth in S7942. Despite the discrepancy in the level of effect on growth in S6803 and S7942, any future use of FapR-based biosensors in either cyanobacteria hosts should include suitable controls and avoid high FapR expression levels, despite its ability to reduce basal biosensor expression.

**Figure 6:**
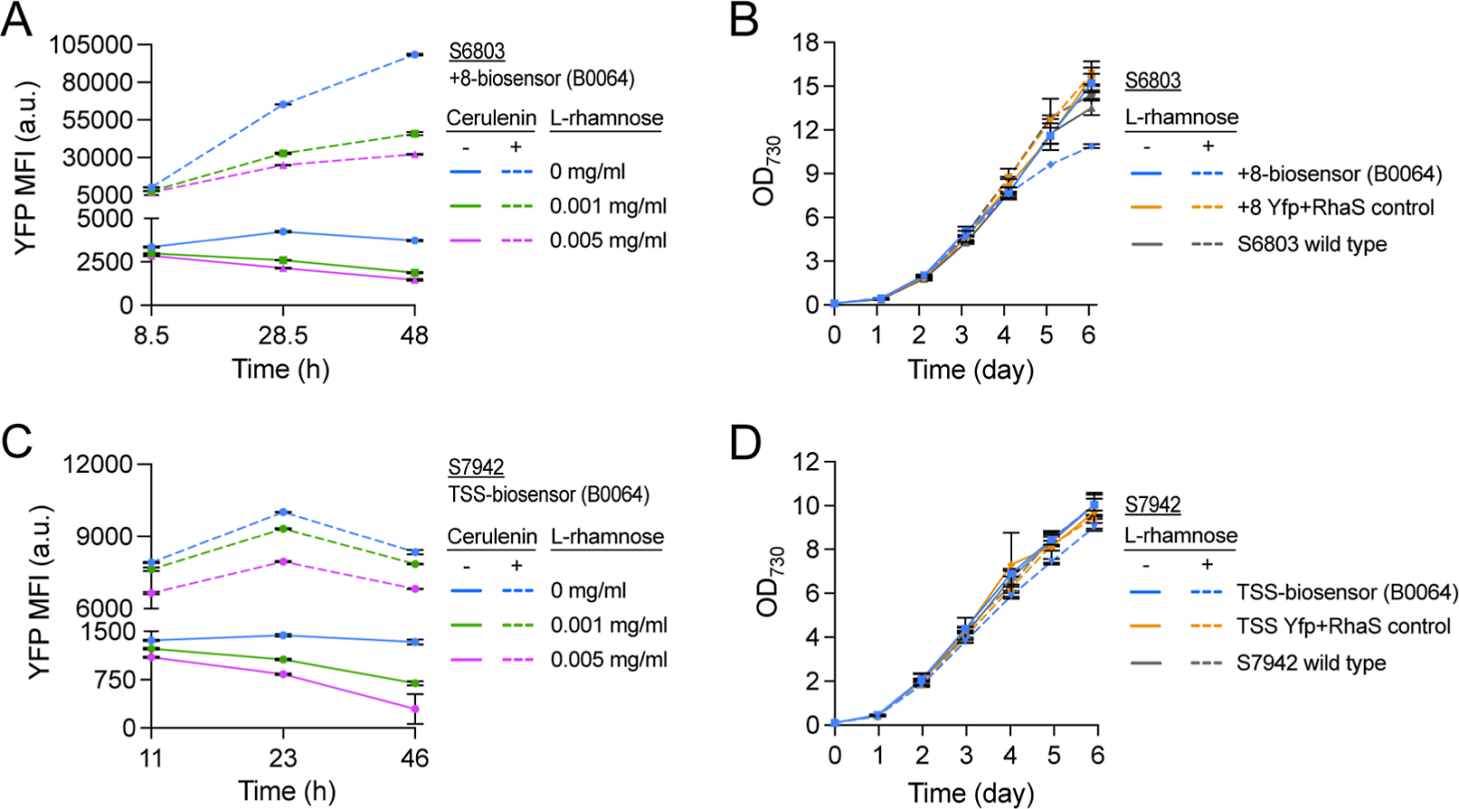
Evaluating biosensor performance at induced FapR levels for selected biosensor constructs in (A,B) S6803 and (C,D) S7942. (A,C) Yfp fluorescence levels measured throughout a two-day time-course; tested biosensors constructs are the (A) + 8-biosensor (P_*rhaBAD*_-B0064) in S6803 and (C) TSS-biosensor (P_*rhaBAD*_-B0064) in S7942. Dashed lines represent the values for biosensor strains treated with 0.5 μg/mL cerulenin; solid lines represent basal (no cerulenin added) biosensor expression. The used concentrations of l-rhamnose to induce P_*rhaBAD*_-B0064-FapR are specified in the figures. Shown Yfp MFI values have been normalized against FITC MFI values obtained for wild-type controls treated with the corresponding cerulenin and/or l-rhamnose concentrations, see the methods section for details. (B–D) Growth curves for selected strains of (B) S6803 and (D) S7942, grown with or without 0.005 mg/mL l-rhamnose, the inducer for P_*rhaBAD*_-B0064-FapR. A wild-type strain and corresponding Yfp + RhaS strain were included as controls. All data is presented as averages ±SD from biological duplicates. Nonvisible error bars are smaller than the data symbol.

## Conclusion

This study describes the establishment of the first malonyl-CoA biosensor for model cyanobacteria S6803 and S7942. This will be useful in applications such as screening of genome-scale libraries produced by, e.g., CRISPR-interference or transposon mutagenesis,^73,74^ to identify which genes are beneficial to target for improving malonyl-CoA levels. If a biosensor application specifically requires low basal expression, then the core-biosensor variant is a suitable option for both S6803 and S7942. If instead a high maximum output signal is required, the +8- or TSS-biosensor variants would be most suited for S6803 and S7942, respectively. The difference between these latter biosensors in terms of the optimal *fapO* operator placement within the FapR-regulated hybrid promoter highlights that top-performing biosensor designs may not be directly transferable between different cyanobacteria strains. However, the results shown herein for S7942 could be applicable to the 99.8% genetically identical UTEX 2973 strain,^39^ thereby expanding the synthetic biology toolset also for this fast-growing strain. Additionally, the more recently characterized fast-growing strains of S11801 and S11802, which are both 83% genetically identical to S7942,^40,41^ have been shown to successfully express S7942-compatible constructs for improved succinic acid and heterologous mannitol bioproduction,^75,76^ indicating that a biosensor that is functional in S7942 could be transferable to these strains as well. Another cyanobacteria strain of interest is the marine *Synechococcus* sp. PCC 11901 (hereafter S11901), which has been successfully engineered to produce the highest free fatty acid yield (∼1.5 g/L) in cyanobacteria.^77^ While S11901 is not closely related to either of the freshwater S6803 and S7942 strains evaluated in this study, the l-rhamnose-inducible P_*rhaBAD*_ and S6803-sourced full-length P_*psbA2*_ promoters used to construct the parts of the established malonyl-CoA biosensor have been shown to both be functional in S11901,^78^ hopefully indicating that the described malonyl-CoA biosensor could be transferred in some capacity and used to further improve the already high free fatty acid yield in this strain.

This study is also the first to evaluate and show that P_*rhaBAD*_ is functional as an l-rhamnose-inducible promoter in S7942; it maintains relatively low basal expression levels while allowing for titratable induced expression. The difference in the relative performance of the characterized P_*rhaBAD*_-RBS constructs in the S6803 and S7942 hosts, with P_*rhaBAD*_-B0064 underperforming more compared to P_*rhaBAD*_-B0034 in S6803 than in S7942, highlights how differently the same RBS parts can perform relative to each other in different cyanobacteria. The included benchmarking of the P_*rhaBAD*_-RBS constructs described in this study to other commonly used promoters in S6803 or S7942 provides a useful reference data set and can help guide the choice of inducer concentration for new applications of the P_*rhaBAD*_ promoter.

Low, basal-level FapR expression from uninduced P_*rhaBAD*_ was enough to enable a working biosensor in both S6803 and S7942. Induced, higher FapR levels helped reduce the basal biosensor Yfp-output but also caused negative growth effects in both S6803 and S7942, indicating a potential lack of orthogonality between FapR and the two cyanobacteria hosts. Therefore, out of the described biosensor sensing modules, the uninduced P_*rhaBAD*_-B0064-FapR sensing module should be favored due to its lower basal expression levels. Future work could aim to replace the P_*rhaBAD*_ system with another weak promoter, ensuring that FapR levels are kept low and thereby also freeing the P_*rhaBAD*_ system for other applications within an engineered strain. Additionally, as the overall Yfp fluorescence levels supported by the P_*rhaBAD*_-RBS and biosensor constructs in S7942 were lower than what was observed in S6803, it is possible that stronger Yfp-output levels could be achieved by expressing these constructs from the replicative pPMQAK1-T vector backbone^47^ also in S7942. Moreover, further optimization of the described biosensor should focus on strategies such as additional hybrid promoter design and engineering of the affinity between FapR and *fapO* in order to improve the biosensor’s dynamic range.

## Materials and Methods

### Strains and General Experimental Conditions

See Table S1 for strains used in this study. Herein, a nonmotile (GT-S derivate) *Synechocystis* sp. PCC 6803 and *Synechococcus elongatus* PCC 7942 were used. Cultivations were done at 30 °C, 1% (v/v) CO_2_, orbital shaking at 150 rpm, and continuous illumination with white light at 50 μmol of photons m^–2^ s^–1^, using a Percival Climatics SE-1100 climate chamber. The BG11-media^79^ was buffered to pH 7.9 with 25 mM HEPES. For growth on solid BG11, 1.5% (w/v) agar and 0.3% (w/v) sodium thiosulfate were added. Antibiotics were supplemented at 50 μg/mL kanamycin, 25 μg/mL chloramphenicol, and 50 μg/mL spectinomycin for S6803 or 17 μg/mL kanamycin for S7942. Cerulenin was added at the indicated concentrations using a 2 mg/mL cerulenin stock (dissolved in absolute ethanol) at the start of the experiments. l-Rhamnose was added when specified at the indicated concentrations using 2 or 6 mg/mL stocks (dissolved in BG11) also at the start of the experiment. All experiments to characterize the Yfp fluorescence levels supported by P_*rhaBAD*_, the P_*psbA2‑fapO*_ hybrid promoters in constructs without or with coexpression of RhaS, and for the full biosensor constructs were performed in 24-deep-well plates (Axygen P-DW-10 ML-24 C, rectangular wells with V-type bottom, 10 mL total volume); these cultures were started at OD_730_ 0.1 in 2.5 mL. The 24-deep-well plates were sealed with sterile gas-permeable adhesive seals (Diversified Biotech BERM-2000), and the plate sides were covered with aluminum foil to allow equal light supply from above to all wells. Samples were taken for flow cytometry analysis at the indicated time-points. Only the growth curve experiments done to evaluate the growth effect of cerulenin on wild-type S6803 and S7942 as well as to assess the growth effect of l-rhamnose induction of P_*rhaBAD*_-B0064-FapR were performed in 50 mL Erlenmeyer flasks; here, cultures were started at OD_730_ 0.1 in 10 mL. Growth was monitored by measuring the OD_730_. Precultures to all experiments were started from biological duplicates or triplicates (as indicated) isolated from solid BG11 plates; these were cultured as indicated above in 24-deep-well plates until they reached an OD_730_ of 3–8.

### Vector Construction

For vectors constructed in this study, see Table S1. Vector maps will be made available at Zenodo. All primers are given in Table S2. All subcloning was done in *Escherichia coli* XL1-Blue or DH5alpha. All constructs were built by standard ligation or Golden Gate assembly. All assembly reactions used Thermo Fisher Scientific T4 DNA ligase, and Golden Gate assemblies also used FastDigest BpiI or BsaI (as specified). The pPMQAK1-T vector used for S6803 constructs already has Golden Gate-compatible BpiI-sites.^47^ For S7942 constructs, Golden Gate-compatible BsaI-sites were added to a pMD19-ΔNS1 backbone by PCR-amplification. Required inserts were PCR-amplified, wherein necessary restriction sites or other sequences were added with primer extensions. Three different terminators were used in the constructed vectors; these were the BioBrick BBa_B0015, ECK120015170, and ECK120034435.^80^ See the Supporting Information for further assembly details, the codon optimized *B. subtilis* FapR sequence, and sequences for the evaluated P_*rhaBAD*_-RBS and P_*psbA2*_/P_*trc*_/P_L22_ promoters.

### Strain Construction

All S6803 strains were created by electroporation; cells from 5 to 10 mL of exponentially growing culture (OD_730_ 0.6–1) were combined with 100–400 ng of vector DNA, and this mixture was electroporated by using setting EC2 (*V* = 2.5 kV) on a Bio-Rad MicroPulser. The electroporated cells were resuspended in a total of 3 mL BG11 and allowed to recover for 16–24 h in a 24-deep-well plate (for cultivation conditions and plate details see above), whereby all cells were collected and concentrated before being plated on selective solid BG11 plates. All S7942 strains were created by natural transformation^81^ in order to integrate constructs into NS1 (Synpcc7942_2498 locus); cells from 5 to 10 mL exponentially growing culture (OD_730_ 1–3) were resuspended in 0.3 mL of BG11 and combined with 1–2 μg vector DNA; this mixture was incubated overnight in darkness combined with otherwise standard cultivation conditions (see above for details), before plating the whole suspension on selective solid BG11 plates. Obtained S7942 colonies were restreaked on fresh selective solid BG11 plates until fully segregated S7942 clones could be confirmed.

### Flow Cytometry, MFI-Value Normalization, and Data Analysis

Culture aliquots taken at indicated time-points were diluted to OD_730_ < 0.1 prior to analysis on a CytoFLEX (Beckman Coulter). Channel PC5.5 (excitation 488 nm, emission 690/50 nm) was used to acquire 10,000 chlorophyll-a-positive events, whereby their Yfp median fluorescence intensity (MFI) was determined in the FITC channel (excitation 488 nm, emission 525/40 nm). See Figure S5a,b for gating examples. Data analysis was done with CytExpert Software (Beckman Coulter) or FlowJo (BD Biosciences). Where indicated, Yfp MFI-values were normalized against wild-type FITC-channel MFI-values by subtracting the average of the relevant wild-type FITC-channel MFI-values from the average of the Yfp MFI-values to be normalized. The standard deviation error bars shown for Yfp MFI values presented in graphs have been calculated by an error propagation formula for subtraction, wherein the standard deviation for the average of the relevant wild-type FITC-channel MFI-values and the standard deviation for the average of the Yfp MFI-values to be normalized have both been taken into consideration. The normalized Yfp MFI-values were used when calculating the ratio or percentage values presented throughout the text; examples where such values were discussed include when calculating the promoter strength percentage of P_*rhaBAD*_-RBS compared to other comparison promoters, the loss in promoter strength for P_*psbA2_tr*_ when combined with *fapO*, the percentage of P_*psbA2_tr‑fapO*_ hybrid promoter repression when coexpressed with FapR, the induction-folds for the biosensor constructs when cells were treated with cerulenin, and the reduction in biosensor Yfp-output in response to l-rhamnose-induced FapR levels.

## Supplementary Material



## Notes

Additional time-course data and comparison to other commonly used promoters for the evaluation of the l-rhamnose-inducible P_*rhaBAD*_ in S6803 and S7942; additional time-course data for the cerulenin-treated biosensor strains and controls in S6803 and S7942; representative plots for flow cytometry gating used in this study; tables of used strains and vectors and primers; sequences of evaluated promoter–RBS combinations (P_*rhaBAD*_, P_*trc*_, P_*psbA2*_, and P_L22_) and the codon-optimized FapR; and a detailed description of the construct assembly (PDF)

## Abbreviations

RBS: ribosome binding site
MFI: median fluorescence intensity
a.u.: arbitrary unit
wt: wild type
NS: neutral site
TSS: transcription start site
CRP: cAMP receptor protein
SD: standard deviation.
