# Supplementary material for: Establishing a Malonyl-CoA Biosensor for the Two Model Cyanobacteria Synechocystis sp. PCC 6803 and Synechococcus elongatus PCC 7942
Source: ACS Synth Biol. 2025 Jun 30;14(7):2865–77. doi: 10.1021/acssynbio.5c00320 (PMC12281612; doi:10.1021/acssynbio.5c00320)
Supplement: Supplementary file 1 [file sb5c00320_si_001.pdf]

Supporting information

Establishing a malonyl-CoA biosensor for the two model cyanobacteria *Synechocystis* sp. PCC 6803 and *Synechococcus elongatus* PCC 7942

Ivana Cengic[a], Elton Paul Hudson[a *]

[a]School of Engineering Sciences in Chemistry, Biotechnology and Health, Science for Life Laboratory, KTH Royal Institute of Technology, 106 91 Stockholm, Sweden

*Corresponding author: huds@kth.se

Contents

- Figure S1-S5
- Table S1-S2
- Sequence of evaluated promoters, RBS, and FapR
- Supporting methods describing detailed construct assembly

Figures

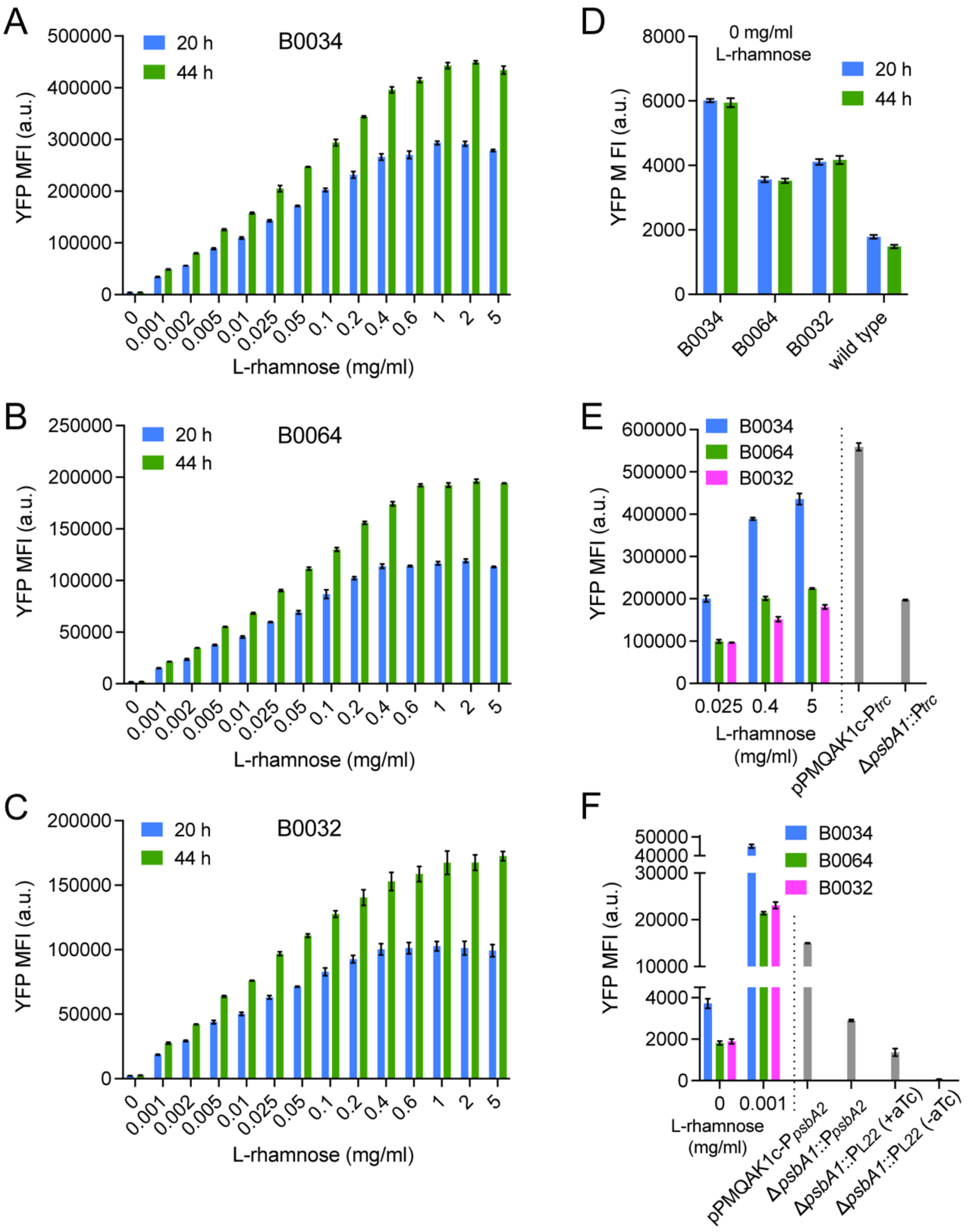

Figure S1. Additional time-course data (day 1-2 of time-course) as well as promoter comparison data for the L-rhamnose inducible P_{rhaBAD} constructs in S6803. (A-C) Yfp fluorescence levels measured at day 1-2 of the time-course for P_{rhaBAD} combined with RBS (A) B0034, (B) B0064, or (C) B0032. Induction was done with 0-5 mg/ml L-rhamnose. Shown Yfp MFI values have been normalized against the FITC MFI values obtained for a wild type control, see the methods section in main manuscript for details. (D) Non-normalized Yfp fluorescence levels at un-induced conditions (0 mg/ml L-rhamnose) at day 1-

2 of the time-course, compared to a wild type control. (E-F) Yfp fluorescence levels measured at time-point 68 h (day 3) of the time-course for comparison promoter constructs and P_{rhaBAD}-constructs induced at specified L-rhamnose concentrations. Shown Yfp MFI values have been normalized against the FITC MFI values obtained for a wild type control, see the methods section in main manuscript for details. Note that the selected shown P_{rhaBAD}-values are the same as in Figure 2b in the main manuscript, these were duplicated here in order to make the comparisons easier to visualize. (E) Comparison to the strong P_{trc}, expressed from a RSF1010-based pPMQAK1 replicative shuttle vector (pPMQAK1c-P_{trc}-B0034) or a genomically integrated construct (Δ*psbA1*::P_{trc}-B0034). In both P_{trc}-constructs the promoter was combined with RBS B0034, see further down in this document for the full P_{trc}-B0034 sequences. (F) Comparison to the moderate P_{psbA2}, from a RSF1010-based pPMQAK1 replicative shuttle vector (pPMQAK1c-P_{psbA2}) or a genomically integrated construct (Δ*psbA1*::P_{psbA2}), as well as from the weak anhydrotetracycline-inducible (aTc) P_{L22} expressed from a genomically integrated construct (Δ*psbA1*::P_{L22}-RBS*). The P_{psbA2}-constructs used the native RBS (AAGGAA), while P_{L22} was combined with RBS*, see further down in this document for the full promoter-RBS sequences. All data is presented as averages ± SD from biological triplicates.

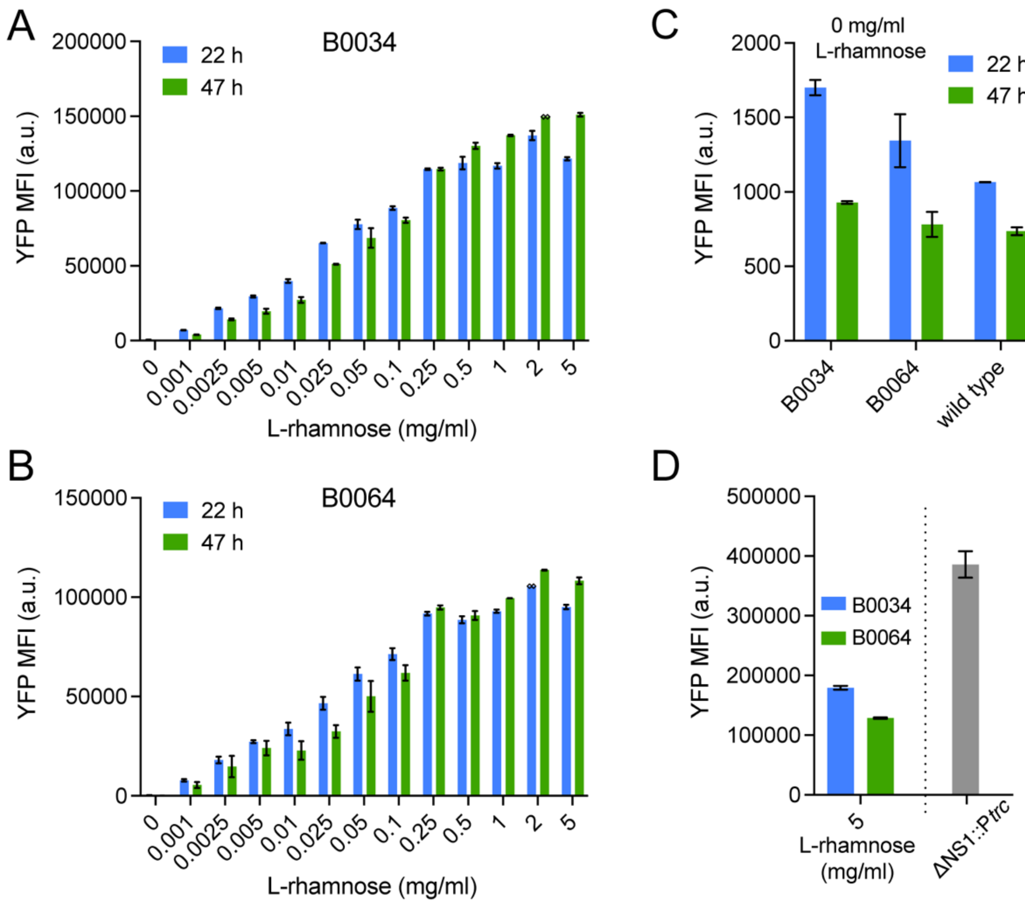

Figure S2. Additional time-course data (day 1-2 of time-course) and promoter comparison data for the L-rhamnose inducible P_{rhaBAD} constructs in S7942. (A-C) Yfp fluorescence levels measured at day 1-2 of the time-course for P_{rhaBAD} combined with RBS (A) B0034 or (B)

B0064. Induction was done with 0-5 mg/ml L-rhamnose. Shown Yfp MFI values have been normalized against the FITC MFI values obtained for a wild type control, see the methods section in main manuscript for details. (C) Non-normalized Yfp fluorescence levels at un-induced conditions (0 mg/ml L-rhamnose) at day 1-2 of the time-course, compared to a wild type control. (D) Comparing the Yfp fluorescence levels measured at time-point 70.5 h (day 3) of the time-course for the genomically integrated (into NS1, i.e. locus Synpcc7942_2498), constitutively expressed P_{trc}-B0034 (ΔNS1::P_{trc}-B0034) comparison promoter construct and P_{rhaBAD}-constructs induced at the maximum tested L-rhamnose concentration (5 mg/ml). Shown Yfp MFI values have been normalized against the FITC MFI values obtained for a wild type control, see the methods section in main manuscript for details. Note that the selected shown P_{rhaBAD}-values are the same as in Figure 2e in the main manuscript, these were duplicated here in order to make the comparisons easier to visualize. All data is presented as averages ± SD from biological duplicates. Individual duplicate values are shown as open diamonds only for the data with non-visible error-bars.

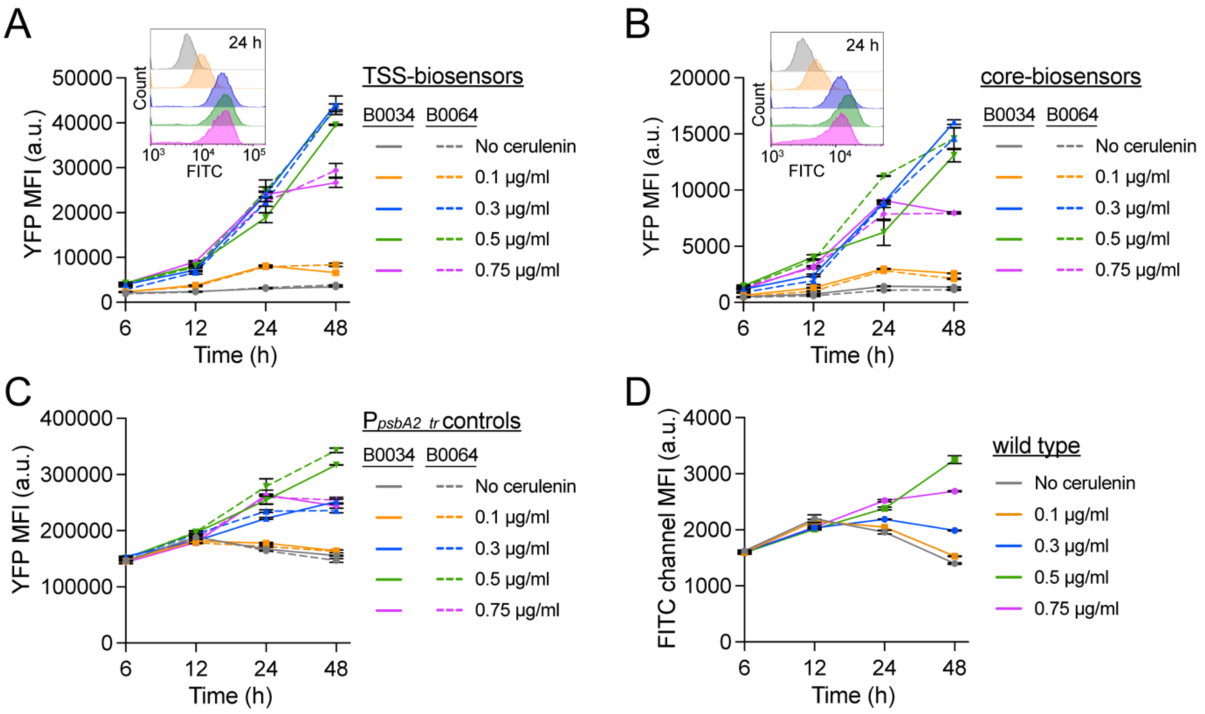

Figure S3. Additional data from evaluating the malonyl-CoA biosensor constructs in S6803. (A-C) Yfp fluorescence levels measured for the (A) TSS-biosensor variants, (B) core-biosensor variants, and (C) P_{psbA2_tr} control constructs (no *fapO*), throughout a two-day time-course of treatment with 0-0.75 µg/ml cerulenin; basal (no cerulenin added) values are also shown. Shown Yfp MFI values have been normalized against FITC MFI values obtained for wild type controls treated with the corresponding cerulenin concentrations, see the methods section in main manuscript for details. P_{rhaBAD}-B0034 and P_{rhaBAD}-B0064 constructs are represented by solid or dashed lines, respectively. The inset figures in (A-B) show overlayed histograms of the FITC channel data (excitation 488 nm, emission 525/40 nm) for the corresponding P_{rhaBAD}-B0064 biosensor at 24 h (the colors are consistent with the legends in the main figures). (D) Time-course data of MFI-values detected in the FITC channel

(excitation 488 nm, emission 525/40 nm) for wild type S6803 treated with 0-0.75 µg/ml cerulenin. All data is presented as averages ± SD from biological duplicates. Non-visible error-bars are smaller than the data symbol.

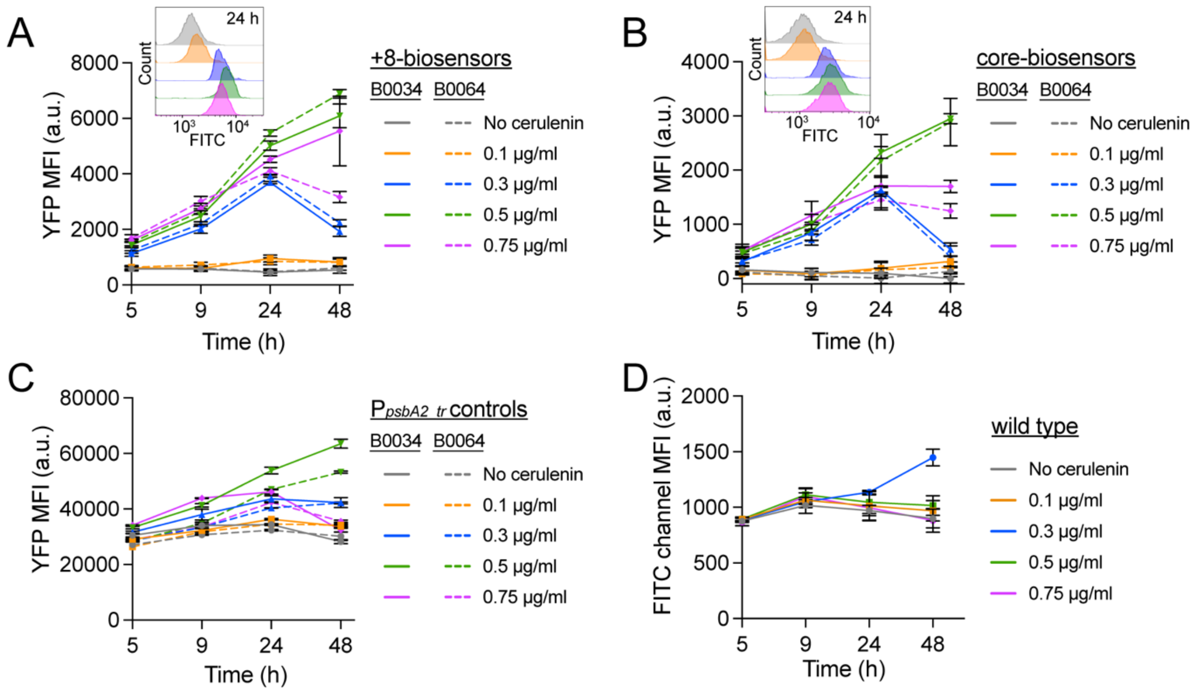

Figure S4. Additional data from evaluating the malonyl-CoA biosensor constructs in S7942. (A-C) Yfp fluorescence levels measured for the (A) +8-biosensor variants, (B) core-biosensor variants, and (C) P_{psbA2_tr} control constructs (no *fapO*), throughout a two-day time-course of of treatment with 0-0.75 µg/ml cerulenin; basal (no cerulenin added) values are also shown. Shown Yfp MFI values have been normalized against FITC MFI values obtained for wild type controls treated with the corresponding cerulenin concentrations, see the methods section in main manuscript for details. P_{rhaBAD}-B0034 and P_{rhaBAD}-B0064 constructs are represented by solid or dashed lines, respectively. The inset figures in (A-B) show overlayed histograms of the FITC channel data (excitation 488 nm, emission 525/40 nm) for the corresponding P_{rhaBAD}-B0064 biosensor at 24 h (the colors are consistent with the legends in the main figures). (D) Time-course data of MFI-values detected in the FITC channel (excitation 488 nm, emission 525/40 nm) for wild type S7942 treated with 0-0.75 µg/ml cerulenin. All data is presented as averages ± SD from biological duplicates. Non-visible error-bars are smaller than the data symbol.

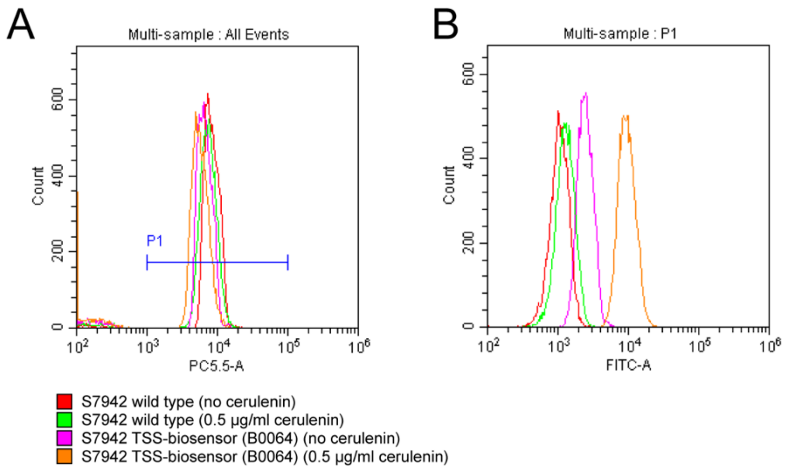

Figure S5. Representative plots showing the gating procedure used within this study. Shown plots are for S7942, but the same applied to S6803. (A) Histogram overlay plot of acquisition in the PC5.5-channel (excitation 488 nm, emission 690/50 nm). Shown are the total events acquired for wild type and TSS-biosensor (B0064) strains of S7942, either when un-treated or treated with 0.5 µg/ml cerulenin. The shown P1 gate was used for collecting 10,000 chlorophyll-a-positive events. (B) The 10,000 events from gate P1 were analyzed within the FITC-channel (excitation 488 nm, emission 525/40 nm) to determine the median fluorescence intensity (MFI) resulting from either *yfp* expression, or background and autofluorescence within this channel. The color legends are the same as in (A).

Tables

Table S1. Strains and vectors used or constructed in this study.

Strain or Vectors	Relevant characteristics	Source
**Strains**		
Escherichia coli XL1-Blue	Cloning host	Stratagene
Escherichia coli DH5alpha	Cloning host	-
Synechocystis sp. PCC 6803	Wild type, non-motile, GT-S derivative	M. Fulda
Synechococcus elongatus PCC 7942	Wild type	ATCC
**Available vectors (in-lab or published)**		
pPMQAK1-T	Replicative vector (RSF1010). Amp^{r}, Kan^{R}.	Vasudevan et al.[1]
pCK314 (Addgene #110545)	Plasmid with P_{rhaBAD} and RhaS, used as PCR-template. Kan^{R}.	Kelly et al.[2]
pMD19-ΔNS1(S7942)::Yfp-B0015-Cm^{R}(rev)	NS1: *Synpcc7942_2498* locus. Cm^{R}. Used as PCR-template.	In-lab. I. Cengic (unpublished)
pPMQAK1c-P_{psbA2}-Yfp-B0015	For comparison test. Replicative vector based on the RSF1010-based pPMQAK1 shuttle vector.[3] P_{psbA2}-Yfp-reporter vector. Amp^{R}, Cm^{R}. P_{psbA2} from S6803.	In-lab. D. Kaczmarzyk (unpublished)
pMD19-Δ*psbA1*(S6803)::P_{psbA2}-Yfp-B0015-Sp^{R}(rev)	For comparison test. S6803 locus *slr1181* (*psbA1*). P_{psbA2}-Yfp-reporter vector. Sp^{R}. P_{psbA2} from S6803.	In-lab. D. Kaczmarzyk (unpublished)
pPMQAK1c-P_{trc}-B0034-Yfp-B0015	For comparison test. Replicative vector based on the RSF1010-based pPMQAK1 shuttle vector.[3] P_{trc}-Yfp-reporter vector, RBS B0034. Amp^{R}, Cm^{R}. No LacI.	In-lab. D. Kaczmarzyk (unpublished)
pMD19-Δ *psbA1*(S6803)::P_{trc}-B0034-Yfp-B0015-Sp^{R}(rev)	For comparison test. S6803 locus *slr1181* (*psbA1*). P_{trc}-Yfp-reporter vector, RBS B0034. Sp^{R}. No LacI.	In-lab. D. Kaczmarzyk (unpublished)
pMD19-Δ*psbA1*(S6803)::P_{J23101}-TetR_LVA-B0015-P_{L22}-RBS*-Yfp-B0015-Sp^{R}(rev)	For comparison test. S6803 locus *slr1181* (*psbA1*). P_{L22}-Yfp-reporter vector, RBS*.	In-lab.

	Sp^R. Anhydrotetracycline-inducible P_{L22}, TetR tagged with C-terminal LVA-tag included.	D. Kaczmarzyk (unpublished)
**Vectors constructed in this study**		
pPMQAK1-P_{rhaBAD}-B0034-Yfp-B0015-P_{J23111}-RhaS-ECK120015170	S6803 P_{rhaBAD}-Yfp-reporter vector, Amp^R, Kan^R. RBS B0034.	This study
pPMQAK1-P_{rhaBAD}-B0064-Yfp-B0015-P_{J23111}-RhaS-ECK120015170	S6803 P_{rhaBAD}-Yfp-reporter vector, Amp^R, Kan^R. RBS B0064.	This study
pPMQAK1-P_{rhaBAD}-B0032-Yfp-B0015-P_{J23111}-RhaS-ECK120015170	S6803 P_{rhaBAD}-Yfp-reporter vector, Amp^R, Kan^R. RBS B0032.	This study
pPMQAK1c-P_{psbA2_tr}-Yfp-B0015	S6803 Yfp-reporter vector, Amp^R, Cm^R. Variant: No *fapO*.	This study
pPMQAK1c-$P_{psbA2\_tr-fapO:+8}$-Yfp-B0015	S6803 Yfp-reporter vector, Amp^R, Cm^R. Variant: *fapO* after +8.	This study
pPMQAK1c-$P_{psbA2\_tr-fapO:core}$-Yfp-B0015	S6803 Yfp-reporter vector, Amp^R, Cm^R. Variant: *fapO* at core.	This study
pPMQAK1c-$P_{psbA2\_tr-fapO:TSS}$-Yfp-B0015	S6803 Yfp-reporter vector, Amp^R, Cm^R. Variant: *fapO* at TSS.	This study
pPMQAK1c-$P_{psbA2\_tr-fapO:core\_+8}$-Yfp-B0015	S6803 Yfp-reporter vector, Amp^R, Cm^R. Variant: *fapO* after +8 and at core.	This study
pPMQAK1c-$P_{psbA2\_tr-fapO:core\_TSS}$-Yfp-B0015	S6803 Yfp-reporter vector, Amp^R, Cm^R. Variant: *fapO* at TSS and core.	This study
pPMQAK1-P_{psbA2_tr}-Yfp(rev)-ECK120034435-P_{J23111}-B0034-RhaS-ECK120015170	S6803 Yfp-reporter + RhaS vector, Amp^R, Kan^R. Variant: No *fapO*.	This study
pPMQAK1-$P_{psbA2\_tr-fapO:+8}$-Yfp(rev)-ECK120034435-P_{J23111}-B0034-RhaS-ECK120015170	S6803 Yfp-reporter + RhaS vector, Amp^R, Kan^R. Variant: *fapO* after +8.	This study
pPMQAK1-$P_{psbA2\_tr-fapO:core}$-Yfp(rev)-ECK120034435-P_{J23111}-B0034-RhaS-ECK120015170	S6803 Yfp-reporter + RhaS vector, Amp^R, Kan^R. Variant: *fapO* at core.	This study
pPMQAK1-$P_{psbA2\_tr-fapO:TSS}$-Yfp(rev)-ECK120034435-P_{J23111}-B0034-RhaS-ECK120015170	S6803 Yfp-reporter + RhaS vector, Amp^R, Kan^R. Variant: *fapO* at TSS.	This study
pPMQAK1-$P_{psbA2\_tr-fapO:core\_+8}$-Yfp(rev)-ECK120034435-$P_{J23111}$-B0034-RhaS-ECK120015170	S6803 Yfp-reporter + RhaS vector, Amp^R, Kan^R. Variant: *fapO* after +8 and at core.	This study
pPMQAK1-$P_{psbA2\_tr-fapO:core\_TSS}$-Yfp(rev)-ECK120034435-$P_{J23111}$-B0034-RhaS-ECK120015170	S6803 Yfp-reporter + RhaS vector, Amp^R, Kan^R. Variant: *fapO* at TSS and core.	This study
pPMQAK1-P_{psbA2_tr}-Yfp(rev)-ECK120034435-P_{rhaBAD}-B0034-FapR-B0015-P_{J23111}-B0034-RhaS-ECK120015170	S6803 Full biosensor construct, Amp^R, Kan^R. Variant: No *fapO,* RBS B0034.	This study
pPMQAK1-$P_{psbA2\_tr-fapO:+8}$-Yfp(rev)-ECK120034435-P_{rhaBAD}-B0034-FapR-B0015-P_{J23111}-B0034-RhaS-ECK120015170	S6803 Full biosensor construct, Amp^R, Kan^R. Variant: *fapO* after +8, RBS B0034.	This study
pPMQAK1-$P_{psbA2\_tr-fapO:core}$-Yfp(rev)-ECK120034435-P_{rhaBAD}-B0034-FapR-B0015-P_{J23111}-B0034-RhaS-ECK120015170	S6803 Full biosensor construct, Amp^R, Kan^R. Variant: *fapO* at core, RBS B0034.	This study
pPMQAK1-$P_{psbA2\_tr-fapO:TSS}$-Yfp(rev)-ECK120034435-P_{rhaBAD}-B0034-FapR-B0015-P_{J23111}-B0034-RhaS-ECK120015170	S6803 Full biosensor construct, Amp^R, Kan^R. Variant: *fapO* at TSS, RBS B0034.	This study
pPMQAK1-$P_{psbA2\_tr-fapO:core\_+8}$-Yfp(rev)-ECK120034435-$P_{rhaBAD}$-B0034-FapR-B0015-$P_{J23111}$-B0034-RhaS-ECK120015170	S6803 Full biosensor construct, Amp^R, Kan^R. Variant: *fapO* after +8 and at core, RBS B0034.	This study
pPMQAK1-$P_{psbA2\_tr-fapO:core\_TSS}$-Yfp(rev)-ECK120034435-$P_{rhaBAD}$-B0034-FapR-B0015-$P_{J23111}$-B0034-RhaS-ECK120015170	S6803 Full biosensor construct, Amp^R, Kan^R. Variant: *fapO* at TSS and core, RBS B0034.	This study
pPMQAK1-P_{psbA2_tr}-Yfp(rev)-ECK120034435-P_{rhaBAD}-B0064-FapR-B0015-P_{J23111}-B0034-RhaS-ECK120015170	S6803 Full biosensor construct, Amp^R, Kan^R. Variant: No *fapO,* RBS B0064.	This study
pPMQAK1-$P_{psbA2\_tr-fapO:+8}$-Yfp(rev)-ECK120034435-P_{rhaBAD}-B0064-FapR-B0015-P_{J23111}-B0034-RhaS-ECK120015170	S6803 Full biosensor construct, Amp^R, Kan^R. Variant: *fapO* after +8, RBS B0064.	This study
pPMQAK1-$P_{psbA2\_tr-fapO:core}$-Yfp(rev)-ECK120034435-P_{rhaBAD}-B0064-FapR-B0015-P_{J23111}-B0034-RhaS-ECK120015170	S6803 Full biosensor construct, Amp^R, Kan^R. Variant: *fapO* at core, RBS B0064.	This study

pPMQAK1-$P_{psbA2\_tr\text{-}fapO:TSS}$-Yfp(rev)-ECK120034435-$P_{rhaBAD}$-B0064-FapR-B0015-$P_{J23111}$-B0034-RhaS-ECK120015170	S6803 Full biosensor construct, Amp^R, Kan^R. Variant: *fapO* at TSS, RBS B0064.	This study
pPMQAK1-$P_{psbA2\_tr\text{-}fapO:core\_+8}$-Yfp(rev)-ECK120034435-$P_{rhaBAD}$-B0064-FapR-B0015-$P_{J23111}$-B0034-RhaS-ECK120015170	S6803 Full biosensor construct, Amp^R, Kan^R. Variant: *fapO* after +8 and at core, RBS B0064.	This study
pPMQAK1-$P_{psbA2\_tr\text{-}fapO:core\_TSS}$-Yfp(rev)-ECK120034435-$P_{rhaBAD}$-B0064-FapR-B0015-$P_{J23111}$-B0034-RhaS-ECK120015170	S6803 Full biosensor construct, Amp^R, Kan^R. Variant: *fapO* at TSS and core, RBS B0064.	This study
pPMQAK1-P_{rhaBAD}-B0034-FapR-B0015-P_{J23111}-B0034-RhaS-ECK120015170	S6803 FapR + RhaS vector,, Amp^R, Kan^R. Variant: RBS B0034. Used as PCR-template.	This study
pPMQAK1-P_{rhaBAD}-B0064-FapR-B0015-P_{J23111}-B0034-RhaS-ECK120015170	S6803 FapR + RhaS vector,, Amp^R, Kan^R. Variant: RBS B0064. Used as PCR-template.	This study
pMD19-ΔNS1::P_{rhaBAD}-B0034-Yfp-B0015-Kan^R(rev)-P_{J23111}-RhaS-ECK120015170	S7942 P_{rhaBAD}-Yfp-reporter vector, Kan^R. RBS B0034.	This study
pMD19-ΔNS1::P_{rhaBAD}-B0064-Yfp-B0015-Kan^R(rev)-P_{J23111}-RhaS-ECK120015170	S7942 P_{rhaBAD}-Yfp-reporter vector, Kan^R. RBS B0064.	This study
pMD19-ΔNS1::P_{rhaBAD}-B0032-Yfp-B0015-Kan^R(rev)-P_{J23111}-RhaS-ECK120015170	S7942 P_{rhaBAD}-Yfp-reporter vector, Kan^R. RBS B0032.	This study
pMD19-ΔNS1::P_{trc}-B0034-Yfp-B0015-Kan^R(rev)	For comparison test. S7942 P_{trc}-Yfp-reporter vector, Kan^R. P_{trc} + RBS B0034. No LacI. NS1: Synpcc7942_2498	This study
pMD19-ΔNS1::P_{psbA2_tr}- Yfp(rev)- ECK120034435-B0015-Kan^R(rev)	S7942 Yfp-reporter vector, Kan^R. Variant: No *fapO*.	This study
pMD19-ΔNS1::$P_{psbA2\_tr\text{-}fapO:+8}$-Yfp(rev)-ECK120034435-B0015-Kan^R(rev)	S7942 Yfp-reporter vector, Kan^R. Variant: *fapO* after +8.	This study
pMD19-ΔNS1::$P_{psbA2\_tr\text{-}fapO:core}$- Yfp(rev)-ECK120034435-B0015-Kan^R(rev)	S7942 Yfp-reporter vector, Kan^R. Variant: *fapO* at core.	This study
pMD19-ΔNS1::$P_{psbA2\_tr\text{-}fapO:TSS}$- Yfp(rev)-ECK120034435-B0015-Kan^R(rev)	S7942 Yfp-reporter vector, Kan^R. Variant: *fapO* at TSS.	This study
pMD19-ΔNS1::$P_{psbA2\_tr\text{-}fapO:core\_+8}$- Yfp(rev)-ECK120034435-B0015-Kan^R(rev)	S7942 Yfp-reporter vector, Kan^R. Variant: *fapO* after +8 and at core.	This study
pMD19-ΔNS1::$P_{psbA2\_tr\text{-}fapO:core\_TSS}$- Yfp(rev)-ECK120034435-B0015-Kan^R(rev)	S7942 Yfp-reporter vector, Kan^R. Variant: *fapO* at TSS and core.	This study
pMD19-ΔNS1::P_{psbA2_tr}- Yfp(rev)- ECK120034435-B0015-Kan^R(rev)-P_{J23111}-B0034-RhaS-ECK120015170	S7942 Yfp-reporter + RhaS vector, Kan^R. Variant: No *fapO*.	This study
pMD19-ΔNS1::$P_{psbA2\_tr\text{-}fapO:+8}$-Yfp(rev)-ECK120034435-B0015-Kan^R(rev)-P_{J23111}-B0034-RhaS-ECK120015170	S7942 Yfp-reporter + RhaS vector, Kan^R. Variant: *fapO* after +8.	This study
pMD19-ΔNS1::$P_{psbA2\_tr\text{-}fapO:core}$- Yfp(rev)-ECK120034435-B0015-Kan^R(rev)-P_{J23111}-B0034-RhaS-ECK120015170	S7942 Yfp-reporter + RhaS vector, Kan^R. Variant: *fapO* at core.	This study
pMD19-ΔNS1::$P_{psbA2\_tr\text{-}fapO:TSS}$- Yfp(rev)-ECK120034435-B0015-Kan^R(rev)-P_{J23111}-B0034-RhaS-ECK120015170	S7942 Yfp-reporter + RhaS vector, Kan^R. Variant: *fapO* at TSS.	This study
pMD19-ΔNS1::$P_{psbA2\_tr\text{-}fapO:core\_+8}$- Yfp(rev)-ECK120034435-B0015-Kan^R(rev)-P_{J23111}-B0034-RhaS-ECK120015170	S7942 Yfp-reporter + RhaS vector, Kan^R. Variant: *fapO* after +8 and at core.	This study
pMD19-ΔNS1::$P_{psbA2\_tr\text{-}fapO:core\_TSS}$- Yfp(rev)-ECK120034435-B0015-Kan^R(rev)-P_{J23111}-B0034-RhaS-ECK120015170	S7942 Yfp-reporter + RhaS vector, Kan^R. Variant: *fapO* at TSS and core.	This study
pMD19-ΔNS1::P_{psbA2_tr}- Yfp(rev)- ECK120034435-P_{rhaBAD}-B0034-FapR-B0015-Kan^R(rev)-P_{J23111}-B0034-RhaS-ECK120015170	S7942 Full biosensor construct, Kan^R. Variant: No *fapO*, RBS B0034.	This study
pMD19-ΔNS1::$P_{psbA2\_tr\text{-}fapO:+8}$-Yfp(rev)-ECK120034435-$P_{rhaBAD}$-B0034-FapR-B0015-Kan^R(rev)-P_{J23111}-B0034-RhaS-ECK120015170	S7942 Full biosensor construct, Kan^R. Variant: *fapO* after +8, RBS B0034.	This study
pMD19-ΔNS1::$P_{psbA2\_tr\text{-}fapO:core}$- Yfp(rev)-ECK120034435-$P_{rhaBAD}$-B0034-FapR-B0015-Kan^R(rev)-P_{J23111}-B0034-RhaS-ECK120015170	S7942 Full biosensor construct, Kan^R. Variant: *fapO* at core, RBS B0034.	This study
pMD19-ΔNS1::$P_{psbA2\_tr\text{-}fapO:TSS}$- Yfp(rev)-ECK120034435-$P_{rhaBAD}$-B0034-FapR-B0015-Kan^R(rev)-P_{J23111}-B0034-RhaS-ECK120015170	S7942 Full biosensor construct, Kan^R. Variant: *fapO* at TSS, RBS B0034.	This study

pMD19-ΔNS1::P$_{psbA2_tr\text{-}fapO:core_+8}$- Yfp(rev)-ECK120034435-P$_{rhaBAD}$-B0034-FapR-B0015-Kan^R(rev)-P$_{J23111}$-B0034-RhaS-ECK120015170	S7942 Full biosensor construct, Kan^R. Variant: *fapO* after +8 and at core, RBS B0034.	This study
pMD19-ΔNS1::P$_{psbA2_tr\text{-}fapO:core_TSS}$- Yfp(rev)-ECK120034435-P$_{rhaBAD}$-B0034-FapR-B0015-Kan^R(rev)-P$_{J23111}$-B0034-RhaS-ECK120015170	S7942 Full biosensor construct, Kan^R. Variant: *fapO* at TSS and core, RBS B0034.	This study
pMD19-ΔNS1::P$_{psbA2_tr}$- Yfp(rev)- ECK120034435-P$_{rhaBAD}$-B0064-FapR-B0015-Kan^R(rev)-P$_{J23111}$-B0034-RhaS-ECK120015170	S7942 Full biosensor construct, Kan^R. Variant: No *fapO*, RBS B0064.	This study
pMD19-ΔNS1::P$_{psbA2_tr\text{-}fapO:+8}$-Yfp(rev)-ECK120034435-P$_{rhaBAD}$-B0064-FapR-B0015-Kan^R(rev)-P$_{J23111}$-B0034-RhaS-ECK120015170	S7942 Full biosensor construct, Kan^R. Variant: *fapO* after +8, RBS B0064.	This study
pMD19-ΔNS1::P$_{psbA2_tr\text{-}fapO:core}$- Yfp(rev)-ECK120034435-P$_{rhaBAD}$-B0064-FapR-B0015-Kan^R(rev)-P$_{J23111}$-B0034-RhaS-ECK120015170	S7942 Full biosensor construct, Kan^R. Variant: *fapO* at core, RBS B0064.	This study
pMD19-ΔNS1::P$_{psbA2_tr\text{-}fapO:TSS}$- Yfp(rev)-ECK120034435-P$_{rhaBAD}$-B0064-FapR-B0015-Kan^R(rev)-P$_{J23111}$-B0034-RhaS-ECK120015170	S7942 Full biosensor construct, Kan^R. Variant: *fapO* at TSS, RBS B0064.	This study
pMD19-ΔNS1::P$_{psbA2_tr\text{-}fapO:core_+8}$- Yfp(rev)-ECK120034435-P$_{rhaBAD}$-B0064-FapR-B0015-Kan^R(rev)-P$_{J23111}$-B0034-RhaS-ECK120015170	S7942 Full biosensor construct, Kan^R. Variant: *fapO* after +8 and at core, RBS B0064.	This study
pMD19-ΔNS1::P$_{psbA2_tr\text{-}fapO:core_TSS}$- Yfp(rev)-ECK120034435-P$_{rhaBAD}$-B0064-FapR-B0015-Kan^R(rev)-P$_{J23111}$-B0034-RhaS-ECK120015170	S7942 Full biosensor construct, Kan^R. Variant: *fapO* at TSS and core, RBS B0064.	This study

Table S2. Primers used in this study.

Name	Sequence (5' to 3')	Description
**Ultramers or oligos used as PCR-templates [a]**		
IVCE836	ATAGGCGCGCCAGCTTTACAAAACTCTCATTAATCCTTTAGACTAAGTTTAGTCAGTTTTAGTACCTAGTCTTAATAAGGAATTATTCTAGACGC	Ultramer: PpsbA2(xtraShort, fapO at +8) + RBS (PpsbA2) + AscI/XbaI
IVCE837	ATAGGCGCGCCAGCTTTACAATTAGTACCTAGTCTTAATAGACTAAGTTTAGTCAGTTTAAGGAATTATTCTAGACGC	Ultramer: PpsbA2(xtraShort, fapO in core) + RBS (PpsbA2) + AscI/XbaI
IVCE838	ATAGGCGCGCCAGCTTTACAATTAGTACCTAGTCTTAATAGACTAAGTTTAGTCAGTTTTAGTACCTAGTCTTAATAAGGAATTATTCTAGACGC	Ultramer: PpsbA2(xtraShort, fapO in core and at +8) + RBS (PpsbA2) + AscI/XbaI
IVCE839	ATAGGCGCGCCAGCTTTACAATTAGTACCTAGTCTTAATAGACTAAGTTTAGTACCTAGTCTTAAGGAATTATTCTAGACGC	Ultramer: PpsbA2(xtraShort, fapO in core and TSS overlap) + RBS (PpsbA2) + AscI/XbaI
IVCE840	ATAGGCGCGCCAGCTTTACAAAACTCTCATTAATCCTTTAGACTAAGTTTAGTACCTAGTCTTAAGGAATTATTCTAGACGC	Ultramer: PpsbA2(xtraShort, fapO overlap TSS) + RBS (PpsbA2) + AscI/XbaI
IVCE841	ATAGGCGCGCCAGCTTTACAAAACTCTCATTAATCCTTTAGACTAAGTTTAGTCAGTTTAAGGAATTATTCTAGACGC	Ultramer: PpsbA2(xtraShort) + RBS (PpsbA2) + AscI/XbaI
IVCE811	**ACAATTTTCGAAAAAACCCGCTTCGGCGGGTTTTTTTATAGCTAAAA**ACCGGTTTCGAATTGACAATTAATCATCGG	fwd - pConII + ECK120015170 (bold overhang)
IVCE816	**CTCGGTACCAAATTCCAGAAAAGAGACGCTGAAAAGCGTCTTTTTTCGTTTTGGTCC**TTTACAGCTAGCTCAGTCCT	fwd - PJ23101 + ECK120034435 (bold overhang)
**For construction of S6803 vectors [a, b]**		
IVCE828	**GCATCTGAAGACACTGCC**TTCATCTTTCCCTGGTTGC	fwd - PrhaBAD (GG, BpiI)
IVCE829	**GCATCTGAAGACACTTCTAGATTTCTCCTCTTT**TTCATTACGACCAGTCT	rev - PrhaBAD + B0034-XbaI (GG, BpiI)
IVCE833	**CCAATGGAAGACAGTTCTAGATTTCCCCTCTTT**TTCATTACGACCAGTCT	rev - PrhaBAD + B0064-XbaI (GG, BpiI)
IVCE835	**ATCGAGGAAGACAGTTCTAGACTTTCCTGTGTGA**TTCATTACGACCAGTCT	rev - PrhaBAD + B0032-XbaI (GG, BpiI)
IVCE803	**GTAGACGAAGACCTAGA**ATGGTATCCAAAGGCGAG	fwd - Yfp-B0015 (GG, BpiI)

IVCE830	**GCATCTGAAGACGTCAA**TTATAAACGCAGAAAGGCCC	rev - Yfp-B0015 (GG, BpiI)
IVCE806	**GACGAAGACTCTCCCTTGACGGCTAGCTCAGTCCTAGGTATAGTGCTAGCAAAGAGGAGAAATACTAG**ATGACCGTATTACATAGTGTGG	fwd - RhaS + PJ23111-B0034 (added in overhang) (GG, BpiI)
IVCE805	**GTAGACGAAGACTC**ATTGCAGAAAGCCATCCC	rev - RhaS (GG, BpiI)
IVCE834	**GGGACGGGATGGCTTTCTGCAATA**AACAATTTTCGAAAAAACCCGC	fwd - ECK120015170 (extension for overlap with RhaS-part)
IVCE832	**GCATCTGAAGACTCTCCC**TTTTAGCTATAAAAAAACCCGCC	rev - ECK120015170 (GG, BpiI)
IVCE831	**GCATCTGAAGACTCA**TTGACGGCTAGCTCAGTC	fwd - PJ23111-B0034-RhaS-ECK120015170 (GG, BpiI)
IVCE390	**CGATCTAGA**ATGCGCCGAAATAAACGCGAACGACAA	fwd - FapR + XbaI
IVCE392	**TATCTGCAG**TTAGGAATGCTTGCTGCGGT	rev - FapR + stop codon + PstI
IVCE875	ATAGGCGCGCCAGCTTTACA	fwd - PpsbA2(xtraShort) + AscI
IVCE876	GCGTCTAGAATAATTCCTTA	rev - PpsbA2(xtraShort) + XbaI
IVCE918	**CGTTCTGAAGACACCCA**TTGACGGCTAGCTCAGT	fwd - PJ23111-RhaS-ECK120015170 (GG, BpiI)
IVCE914	**CGTTCTGAAGACGTTGCC**GGACCAAAACGAAAAAAGAC	fwd - ECK120034435(rev) (GG, BpiI)
IVCE920	**GGACGAGTTGTATAAGTAG**CTCGGTACCAAATTCCAGA	rev - ECK120034435(rev), for overlap with PpsbA2(xtrashort, fapO variants)-eYFP(rev)
IVCE919	**CTGGAATTTGGTACCGAG**CTACTTATACAACTCGTCCATG	fwd - PpsbA2(xtrashort, fapO variants)-eYFP(rev), for overlap with ECK120034435(rev)
IVCE917	**CGTTCTGAAGACCAAT**GGGCGCGCCAGCTTTACA	rev - PpsbA2(xtrashort, fapO variants)-eYFP(rev) (GG, BpiI)
IVCE926	**AAGTCCGAAGACTC**TTCATCTTTCCCTGGTTGC	fwd - PrhaBAD-FapR-B0015-PJ23111-RhaS-ECK120015170 (GG, BpiI)
IVCE925	**AAGTCCGAAGACCTTGAA**GGGCGCGCCAGCTTTACA	rev - PpsbA2(xtrashort, fapO variants)-eYFP(rev) (GG, BpiI)
**For construction of S7942 vectors [a, b]**		
IVCE1131	**TACGTCGGTCTCGAA**AGTCCCTGCTCGTCACGC	rev - pMD19(NS1, S7942) backbone (GG, BsaI)
IVCE1103	**AACCTGGGTCTCCAA**CCTCCGCCAGCCGTTGGG	fwd - pMD19(NS1, S7942) backbone (GG, BsaI)
IVCE1107	**AACCTGGGTCTCGA**AGGCTCTGCCAGTGTTAC	fwd - KanR (rev) (GG, BsaI)
IVCE1108	**AACCTGGGTCTCGT**TACAACAAAGCCACGTTG	rev - KanR (rev) (GG, BsaI)
IVCE1109	**AACCTGGGTCTCCGTAA**TTGACGGCTAGCTCAGT	fwd - PJ23111-RhaS-ECK120015170 (GG, BsaI)
IVCE1110	**AACCTGGGTCTCCGG**TTTTAGCTATAAAAAAACCCGCC	rev - ECK120015170 (GG, BsaI)
IVCE1130	**TACGTCGGTCTCGCT**TTCATCTTTCCCTGGTTGC	fwd - PrhaBAD (GG, BsaI)
IVCE1106	**AACCTGGGTCTCGCC**TTATAAACGCAGAAAGGCCCC	rev - ECK120034435-YFP-PpsbA2-PrhaBAD-FapR-B0015 (GG, BsaI)
IVCE1105	**AACCTGGGTCTCGCT**GGACCAAAACGAAAAAAGAC	fwd - ECK120034435-YFP-PpsbA2-PrhaBAD-FapR-B0015 (GG, BsaI)
IVCE1111	**GGTTCTGGTCTCCTA**GGGCGCGCCAGCTTTACA	rev - ECK120034435-YFP-PsbA2 (GG, BsaI)
IVCE1112	**GGTTCTGGTCTCCCC**TATAAACGCAGAAAGGCCC	fwd - B0015(rev) (GG, BsaI)
IVCE1113	**GGTTCTGGTCTCCCT**CCAGGCATCAAATAAAACGA	rev - B0015(rev) (GG, BsaI)
IVCE1114	**GGTTCTGGTCTCCGG**AGGCTCTGCCAGTGTTAC	fwd - KanR(rev) (GG, BsaI)
IVCE1116	**CTGAACGGTCTCCGG**TACAACAAAGCCACGTTG	rev - KanR(rev) (GG, BsaI)
IVCE1104	**AACCTGGGTCTCCCC**AGTCCCTGCTCGTCACGC	rev - pMD19(NS1, S7942) backbone (GG, BsaI)
IVCE1115	**CTGAACGGTCTCGTA**CCTCCGCCAGCCGTTGGG	fwd - pMD19(NS1, S7942) backbone (GG, BsaI)
IVCE1525	**CTCAACGGTCTCCGAATTGTGAGCGGATAACAATTTCACACAAAAGAGGAGAAATCTAGA**ATGGTATCCAAAGGCGAGG	fwd - eYFP-KanR, partial Ptrc-B0034 in overhang (GG, BsaI)

IVCE1526	**CTCAACGGTCTCCGG**TTACAACAAAGCCACGTTGT	rev - eYFP-KanR (GG, BsaI)
IVCE1527	**CTCAACGGTCTCGAA**CCTCCGCCAGCCGTTG	fwd - pMD19(NS1, S7942) backbone (GG, BsaI)
IVCE1528	**CTCAACGGTCTCGATTCCACACATTATACGAGCCGGATGATTAATTGTCAACAGCTCAT**AGTCCCTGCTCGTCACGC	rev - pMD19(NS1, S7942) backbone, partial Ptrc-B0034 in overhang (GG, BsaI)

[a] Restriction enzyme sites are underlined and the corresponding restriction enzyme is indicated in the "Description" column.
[b] Bold typeface indicates oligo overhangs, often used for Golden Gate assembly or overlap-PCR.

Sequences

P_{rhaBAD}-B0034/B0064/B0032

TTCATCTTTCCCTGGTTGCCAATGGCCCATTTTCCTGTCAGTAACGAGAAGGTCGCGTATTCAGGCGCTTTTTAGACTGGTCGTAATGAA-**RBS**-TCTAGAATG (start codon underlined, for **RBS** sequences see below)

B0034-AAAGAGGAGAAA
B0064-AAAGAGGGGAAA
B0032-TCACACAGGAAAG

P_{psbA2_tr} (truncated version of P_{psbA2})

See Figure 3a in main text.

$P_{psbA2\_tr\text{-}fapO}$ hybrid promoters

See Figure 3b in main text.

P_{trc}

ATGAGCTGTTGACAATTAATCATCCGGCTCGTATAATGTGTGGAATTGTGAGCGGATAACAATTTCACACA

The above P_{trc}-sequence was combined with the following appended 3'-sequence in the specified comparison constructs:

- S6803 pPMQAK1c-P_{trc}-B0034-Yfp-B0015: **AAAGAGGAGAAA**TCTAGAATG (**RBS B0034**, XbaI, start codon)
- S6803 pMD19-Δ*psbA1*:: P_{trc}-B0034-Yfp-B0015: **AAAGAGGAGAAA**ACTAGATG (**RBS B0034**, start codon)
- S7942 pMD19-ΔNS1:: P_{trc}-B0034-Yfp-B0015: **AAAGAGGAGAAA**TCTAGAATG (**RBS B0034**, XbaI, start codon)

P_{psbA2} in S6803 promoter comparison constructs

AACTGACTGACCACTGACCTTAAGAGTAATGGCGTGCAAGGCCCAGTGATCAATTTCATTATTTTTCATTATTTCATCTCCATTGTCCCTGAAAATCAGTTGTGTCGCCCCTCTACACAGCCCAGAACTATGGTAAAGGCGCACGAAAAACCGCCAGGTAAACTCTTCTCAACCCCCAAAACGCCCTCTGTTTACCCATGGAAAAAACGACAATTACAAGAAAGTAAAACTTATGTCATCTATAAGCTTCGTGTATATTAACTTCCTGTTACAAAGCTTTACAAAACTCTCATTAATCCTTTAGACTAAGTTTAGTCAGTTCCAATCTGAACATCGACAAATACAT**AAGGAA**TTAT (**native P_{psbA2} RBS**)

The above P_{psbA2}-sequence was combined with the following appended 3'-sequence in the specified comparison constructs:

- pPMQAK1c-P_{psbA2}-Yfp-B0015: TCTAGATG (XbaI, start codon)
- pMD19-Δ*psbA1*:: P_{psbA2}-Yfp-B0015: TACTAGATG (start codon)

P_{L22}

TCCCTATCAGTGATAGAGATTGACATCCCTATCAGTGATAGAGATACTGGGAGCTACTAGAG**TAG TGGAGGT**TACTAG<u>ATG</u> (**RBS*** [4], <u>start codon</u>)

Same P_{L22}-construct design as used in a study that developed a CRISPRi-tool for S6803,[5] dCas9 was exchanged for Yfp to create the promoter comparison construct used in this study (P_{J23101}-B0034-TetR_LVA-B0015-P_{L22}-RBS*-Yfp-B0015).

***Bacillus subtilis* FapR gBlock sequence, codon optimized for S6803 with IDT tool**

ATGCGCCGAAATAAACGCGAACGACAAGAATTACTCCAGCAAACTATCCAGGCCACGCCTTTTAT
TACAGACGAAGAATTGGCAGGTAAATTCGGCGTATCTATCCAGACTATCCGACTCGATCGCCTAGA
GCTGTCTATCCCGGAGCTCCGCGAACGTATTAAGAATGTCGCTGAAAAGACTCTGGAGGATGAGG
TGAAGTCTCTAAGTTTAGATGAAGTAATCGGCGAAATCATTGACCTAGAATTGGACGACCAAGCG
ATCTCTATTCTGGAAATCAAACAAGAGCACGTCTTTTCCCGAAACCAAATTGCGCGCGGCCACCAT
CTATTCGCGCAAGCAAACTCCTTAGCCGTCGCCGTCATTGACGACGAGTTGGCCTTGACGGCCAGT
GCAGACATCCGATTTACCCGACAAGTTAAACAGGGTGAACGTGTAGTAGCCAAAGCCAAAGTCAC
AGCTGTCGAGAAGGAAAAAGGTCGGACCGTGGTGGAGGTCAATAGTTATGTCGGCGAGGAAATTG
TTTTCTCTGGACGATTTGATATGTACCGCAGCAAGCATTCC

Supporting methods - detailed construct assembly description

The following section describes in detail the preparation of backbone and insert-parts to assemble the indicated constructs.

<u>S6803 P_{rhaBAD}-RBS-Yfp constructs</u>

pPMQAK1-P_{rhaBAD}-B0034/B0064/B0032-Yfp-B0015-P_{J23111}-B0034-RhaS-ECK120015170

Golden Gate assembly with BpiI into pPMQAK1-T.

- <u>P_{rhaBAD}-RBS variants</u>. Universal forward primer IVCE828. Separate reverse primers add XbaI and different RBS: IVCE829 for B0034, IVCE833 for B0064, and IVCE835 for B0032. Template used: pCK314.
- <u>Yfp-B0015</u>. IVCE803 and IVCE830. Template used: pPMQAK1c-P_{psbA2}-Yfp-B0015.
- <u>P_{J23111}-B0034-RhaS-ECK120015170</u>. Overlap-PCR done with IVCE831-832 to join the following separately amplified parts:
  - <u>P_{J23111}-B0034-RhaS</u>. IVCE805-806, forward IVCE806 adds P_{J23111}-B0034**.** Template used: pCK314.
  - <u>ECK120015170</u>. IVCE834 and IVCE832. Oligo template used: IVCE811.

<u>S6803 $P_{psbA2\_tr\text{-}fapO}$-Yfp-only constructs</u>

pPMQAK1c-$P_{psbA2\_tr\text{-}fapO}$-Yfp-B0015

Standard restriction enzyme digestion followed by ligation assembly.

- <u>$P_{psbA2\_tr\text{-}fapO}$ variants</u>. IVCE875-876, PCR-products digested with AscI-XbaI. Templates used: Ultramers IVCE836-841.
- <u>pPMQAK1c-Yfp-B0015 backbone</u>. Digest a pPMQAK1c-P_{psbA2}-Yfp-B0015 vector with AscI-XbaI to remove promoter.

S6803 $P_{psbA2\_tr\text{-}fapO}$-Yfp + RhaS constructs

pPMQAK1-$P_{psbA2\_tr\text{-}fapO}$-Yfp-ECK120034435-P_{J23111}-B0034-RhaS-ECK120015170

Golden Gate assembly with BpiI into pPMQAK1-T.

- $P_{psbA2\_tr\text{-}fapO}$-Yfp-ECK120034435 variants. Overlap-PCR done with IVCE914 and IVCE917 to join the following separately amplified parts:
  - $P_{psbA2\_tr\text{-}fapO}$-Yfp variants. IVCE919 and IVCE917. Templates used: constructed S6803 $P_{psbA2\_tr\text{-}fapO}$-Yfp-only vectors.
  - ECK120034435. IVCE914 and IVCE920. Oligo template used: IVCE816.
- P_{J23111}-B0034-RhaS-ECK120015170. IVCE918 and IVCE832. Templates used: constructed S6803 P_{rhaBAD}-RBS-Yfp vectors.

S6803 P_{rhaBAD}-RBS-FapR + RhaS constructs

pPMQAK1-P_{rhaBAD}-B0034/B0064-FapR-B0015-P_{J23111}-B0034-RhaS-ECK120015170

Standard restriction enzyme digestion followed by ligation assembly. FapR was sub-cloned into the P_{rhaBAD}-B0034/B0064 expression vectors in order to be used as PCR-templates for insert-amplification for the full biosensor construction.

- FapR. IVCE390 and IVCE392, PCR-product digested with XbaI-PstI. Template used: FapR gBlock.
- pPMQAK1-P_{rhaBAD}-RBS-B0015-P_{J23111}-B0034-RhaS-ECK120015170 backbone. Digested constructed S6803 P_{rhaBAD}-RBS-Yfp vectors with XbaI-PstI to remove the Yfp.

S6803 full biosensor constructs

pPMQAK1-$P_{psbA2\_tr\text{-}fapO}$-Yfp-ECK120034435-P_{rhaBAD}-B0034/B0064-FapR-B0015-P_{J23111}-B0034-RhaS-ECK120015170

Golden Gate assembly with BpiI into pPMQAK1-T.

- $P_{psbA2\_tr\text{-}fapO}$-Yfp-ECK120034435. IVCE914 and IVCE925. Templates used: constructed S6803 $P_{psbA2\_tr\text{-}fapO}$-Yfp + RhaS vectors.
- P_{rhaBAD}-RBS-FapR-B0015-P_{J23111}-B0034-RhaS-ECK120015170. IVCE926 & IVCE832. Templates used: constructed S6803 P_{rhaBAD}-RBS-FapR vectors.

S7942 P_{rhaBAD}-RBS-Yfp constructs

pMD19-ΔNS1::P_{rhaBAD}-B0034/B0064-Yfp-B0015-Kan^R(rev)-P_{J23111}-B0034-RhaS-ECK120015170

Golden Gate assembly with BsaI into amplified pMD19-ΔNS1 backbone.

- pMD19-ΔNS1 backbone. IVCE1131 and IVCE1103. Template used: pMD19-ΔNS1::Yfp-B0015-Cm^R(rev).
- P_{rhaBAD}-RBS-Yfp-B0015 variants. IVCE1130 and IVCE1106. Templates used: constructed S6803 P_{rhaBAD}-RBS-Yfp vectors.
- Kan^R. IVCE1107-1108. Template used: pPMQAK1-T vector.

- P$_{J23111}$-B0034-RhaS-ECK120015170. IVCE1109-1110. Template used: constructed S6803 P$_{rhaBAD}$-RBS-Yfp vector.

S7942 ΔNS1::P$_{trc}$-Yfp comparison constructs
pMD19-ΔNS1::P$_{trc}$-B0034-Yfp-B0015-Kan^{R}(rev)

Golden Gate assembly with BsaI into amplified pMD19-ΔNS1 backbone.

- pMD19-ΔNS1 backbone. IVCE1527-1528, reverse IVCE1528 adds part of P$_{trc}$. Template used: pMD19-ΔNS1::Yfp-B0015-Cm^{R}(rev).
- P$_{trc}$-B0034-Yfp-B0015-Kan^{R}(rev). IVCE1525-1526, forward IVCE1525 adds part of P$_{trc}$ and RBS B0034. Template used: constructed S7942 pMD19-ΔNS1::P$_{rhaBAD}$-RBS-Yfp vector.

S7942 P$_{psbA2_tr\text{-}fapO}$-Yfp-only constructs
pMD19-ΔNS1::P$_{psbA2_tr\text{-}fapO}$-Yfp(rev)-ECK120034435-B0015-Kan^{R}(rev)

Golden Gate assembly with BsaI into amplified pMD19-ΔNS1 backbone.

- pMD19-ΔNS1 backbone. IVCE1104 and IVCE1115. Template used: pMD19-ΔNS1::Yfp-B0015-Cm^{R}(rev).
- P$_{psbA2_tr\text{-}fapO}$-Yfp-ECK120034435. IVCE1105 and IVCE1111. Templates used: constructed S6803 P$_{psbA2_tr\text{-}fapO}$-Yfp + RhaS vectors.
- Kan^{R}. IVCE1114 and IVCE1116. Template used: pPMQAK1-T vector.
- B0015. IVCE1112-1113. Template used: pMD19-ΔNS1::Yfp-B0015-Cm^{R}(rev).

S7942 P$_{psbA2_tr\text{-}fapO}$-Yfp + RhaS constructs
pMD19-ΔNS1::P$_{psbA2_tr\text{-}fapO}$-Yfp(rev)-ECK120034435-B0015-Kan^{R}(rev)-P$_{J23111}$-B0034-RhaS-ECK120015170

Golden Gate assembly with BsaI into amplified pMD19-ΔNS1 backbone.

- pMD19-ΔNS1 backbone. IVCE1103-1104. Template used: pMD19-ΔNS1::Yfp-B0015-Cm^{R}(rev).
- P$_{psbA2_tr\text{-}fapO}$-Yfp-ECK120034435. IVCE1105 and IVCE1111. Templates used: constructed S6803 P$_{psbA2_tr\text{-}fapO}$-Yfp + RhaS vectors.
- Kan^{R}(rev)-P$_{J23111}$-B0034-RhaS-ECK120015170. IVCE1114 and IVCE1110. Template used: Template used: constructed S7942 pMD19-ΔNS1::P$_{rhaBAD}$-RBS-Yfp vector.
- B0015. IVCE1112-1113. Template used: pMD19-ΔNS1::Yfp-B0015-Cm^{R}(rev).

S7942 full biosensor constructs
pMD19-ΔNS1::P$_{psbA2_tr\text{-}fapO}$-Yfp(rev)-ECK120034435-P$_{rhaBAD}$-B0034/B0064-FapR-B0015-Kan^{R}(rev)-P$_{J23111}$-B0034-RhaS-ECK120015170

Golden Gate assembly with BsaI into amplified pMD19-ΔNS1 backbone.

- pMD19-ΔNS1 backbone. IVCE1103-1104. Template used: pMD19-ΔNS1::Yfp-B0015-Cm^{R}(rev).

- $P_{psbA2\ tr\text{-}fapO}$-Yfp-ECK120034435-$P_{rhaBAD}$-B0034/B0064-FapR-B0015. IVCE1105-1106. Templates used: constructed S6803 Full biosensor vectors.
- Kan^{R}(rev)-P_{J23111}-B0034-RhaS-ECK120015170. IVCE1107 and IVCE1110. Template used: constructed S7942 pMD19-ΔNS1::P_{rhaBAD}-RBS-Yfp vector.

References

(1) Vasudevan, R., Gale, G. A. R., Schiavon, A. A., Puzorjov, A., Malin, J., Gillespie, M. D., Vavitsas, K., Zulkower, V., Wang, B., Howe, C. J., Lea-Smith, D. J., and McCormick, A. J. (2019) CyanoGate: A Modular Cloning Suite for Engineering Cyanobacteria Based on the Plant MoClo Syntax. *Plant Physiol 180*, 39–55.

(2) Kelly, C. L., Taylor, G. M., Hitchcock, A., Torres-Méndez, A., and Heap, J. T. (2018) A Rhamnose-Inducible System for Precise and Temporal Control of Gene Expression in Cyanobacteria. *ACS Synth Biol 7*, 1056–1066.

(3) Huang, H.-H., Camsund, D., Lindblad, P., and Heidorn, T. (2010) Design and characterization of molecular tools for a Synthetic Biology approach towards developing cyanobacterial biotechnology. *Nucleic Acids Res 38*, 2577–93.

(4) Heidorn, T., Camsund, D., Huang, H.-H., Lindberg, P., Oliveira, P., Stensjö, K., and Lindblad, P. (2011) Synthetic biology in cyanobacteria engineering and analyzing novel functions. *Methods Enzymol 497*, 539–79.

(5) Yao, L., Cengic, I., Anfelt, J., and Hudson, E. P. (2016) Multiple Gene Repression in Cyanobacteria Using CRISPRi. *ACS Synth Biol 5*, 207–12.
